# Arrhythmogenic influence of mutations in a myocyte-based computational model of the pulmonary vein sleeve

**DOI:** 10.1038/s41598-022-11110-1

**Published:** 2022-04-29

**Authors:** Karoline Horgmo Jæger, Andrew G. Edwards, Wayne R. Giles, Aslak Tveito

**Affiliations:** 1 Simula Research Laboratory, Oslo, Norway; 2 Department of Physiology and Pharmacology, Cumming School of Medicine, University of Calgary, Calgary, Canada

**Keywords:** Computational biology and bioinformatics, Computational models, Computational biophysics

## Abstract

In the heart, electrophysiological dysregulation arises from defects at many biological levels (from point mutations in ion channel proteins to gross structural abnormalities). These defects disrupt the normal pattern of electrical activation, producing ectopic activity and reentrant arrhythmia. To interrogate mechanisms that link these primary biological defects to macroscopic electrophysiologic dysregulation most prior computational studies have utilized either (i) detailed models of myocyte ion channel dynamics at limited spatial scales, or (ii) homogenized models of action potential conduction that reproduce arrhythmic activity at tissue and organ levels. Here we apply our recent model (EMI), which integrates electrical activation and propagation across these scales, to study human atrial arrhythmias originating in the pulmonary vein (PV) sleeves. These small structures initiate most supraventricular arrhythmias and include pronounced myocyte-to-myocyte heterogeneities in ion channel expression and intercellular coupling. To test EMI’s cell-based architecture in this physiological context we asked whether ion channel mutations known to underlie atrial fibrillation are capable of initiating arrhythmogenic behavior via increased excitability or reentry in a schematic PV sleeve geometry. Our results illustrate that EMI’s improved spatial resolution can directly interrogate how electrophysiological changes at the individual myocyte level manifest in tissue and as arrhythmia in the PV sleeve.

## Introduction

Atrial fibrillation (AF) is the most common arrhythmia in adult humans^1,2^. Several ion channel mutations, along with a range of other genetic variants and broader risk factors, are known to increase the likelihood of developing AF^3–5^. The lifetime risk of developing AF after the age of 55 is astonishingly high (37 %, see^6^). Treatment of AF remains a significant challenge^7,8^, perhaps because the biophysical processes initiating and maintaining AF remain unclear^1^. Numerical computations have aided in developing our present understanding of the atrial action potential both for a single atrial myocyte^9–11^ and in tissue^12–17^.

It is well established that AF often is initiated in the pulmonary vein ‘sleeves’ of the left atrium^18–20^, where thin layers of myocardium merge with the fibrous tissue of the pulmonary veins. The densities of certain ion channels expressed in the sarcolemma of myocytes in this region are distinctly different from the rest of the atria^21–24^ and the myocyte-to-myocyte electrical coupling through gap junctions can vary significantly^12,20,25^.

The mathematical approaches commonly used for simulating electrical activity in atrial tissue are the bidomain or monodomain models^12,14,26,27^. However, both of these models require spatial averaging over hundreds of cells and it is therefore not possible to model the significant cell-to-cell variations in the electrophysiological properties that are characteristic of the cardiac myocytes located in the ‘sleeve’ of tissue at the transition from the pulmonary vein to the left atria. In order to allow for cell-to-cell variation in ion channel densities and assess the effects of random variation in gap junction coupling between the myocytes, we have applied our recently developed mathematical model^28–32^ that represents each myocyte individually. This is referred to as the EMI model since it explicitly represents the extracellular (E) space, the membrane (M) and the intracellular (I) space, and thus follows the modeling tradition represented by, e.g.,^25,33^. The average mesh length applied in the EMI model is $\, \Delta x=10\;\mu$m whereas $\, \Delta x=0.25$ mm is the standard mesh length for the bidomain model; see, e.g.,^28,32,34–38^. Consequently, the size of one mesh block is about 1 pL for the EMI model and 15600 pL for the bidomain model. This should be compared to 16 pL which is the volume of an atrial myocyte used in the computations below. This resolution allows EMI to interrogate physiological properties at another scale than is possible with the bidomain model, but at the cost of a 15600-fold increase in the number of mesh blocks. Note that since convergence of the bidomain model is achieved at the mesh size $\sim \!\!\!\, \Delta x=0.25$ mm, further physiological details cannot be achieved by decreasing the mesh parameter using this model. Further comparisons of the bidomain model and the EMI model are found in^38^.

A range of genetic variants and mutations are known to increase the probability of developing AF, and a number of these occur in genes encoding the major cardiac ion channels^3–5^. In some cases, the electrophysiologic outcomes of these mutations have been well characterized, and provide a basis for asking whether a computational framework can be used to identify and characterize clinically meaningful arrhythmogenesis in the pulmonary vein sleeve. To implement such a framework using the EMI model, adequate data sets are available for the following mutations: N588K^39–41^, A545P^42^, E229V^43^, E375X^44^, A130V^45^, c.932delC^46^. Here, we investigate how each of these mutations change the likelihood of maintaining re-entry in a model of the specialized myocytes located at the transition from the pulmonary vein (PV) to the left atrial myocardium (LA). Furthermore, we examine how each mutation can alter the excitability of quiescent tissue in this region, and thus change the probability of triggering ectopic beats. In this numerical model, we introduce electrophysiologic heterogeneity in two ways: (1) by allowing each myocyte to be defined as a random combination of previously measured electrophysiologic phenotypes of PV and LA myocytes, and (2) by allowing electrical coupling to vary randomly among adjacent myocytes.

Our computations reveal that the effect of the selected mutations on the biomarkers of the action potential and cytosolic [$\hbox {Ca}^{2+}$] varies significantly. None of the mutations markedly alter the resting membrane potential (RMP), whereas most have significant effects on the maximal velocity of the action potential (AP) upstroke, and AP duration (APD). As expected, we observed re-entry for a range of mutations that either slowed conduction velocity or shortened APD, namely N588K, E299V, A130V and c.932delC. Re-entry was not observed for A545P or E375X (and not for the baseline WT model). Tissue excitability was unchanged for most mutations, but significantly decreased for A130V, and significantly increased for c.932delC. Together, these results suggest that this high resolution (EMI) modeling approach can be applied to simulating the heterogeneous PV-LA junction, and thereby identify and discriminate the arrhythmogenic influence of clinically meaningful perturbations to myocyte electrophysiology.

## Methods

In this section, we describe the models used in our simulations of the PV sleeve. First, we describe membrane models used to account for the ionic currents and intracellular ion concentrations of LA and PV cardiomyocytes. Next, we explain how each of the six considered mutations are represented in the models and the setup used for the finite element simulations of the EMI model. Finally, we provide the definitions of the biomarkers used in our computations.

### Membrane models for PV and LA cardiomyocytes

To represent the resting membrane potential and action potential of both human LA and PV cardiomyocytes (CMs), we have utilized adjusted versions of the previously published base model^40,47^. Versions of our model have previously been used to simulate human induced pluripotent stem cell-derived CMs (hiPSC-CMs) and healthy adult human, canine, rabbit, guinea pig and zebrafish ventricular CMs^47,48^. In addition, this formalism has been used to study the short QT syndrome mutation N588K in ventricular CMs^40,49^. The base model used in this study is similar to the version used in^40,49^. Additional membrane currents (e.g., $I_{\mathrm {Kur}}$) have been added to more accurately represent the AP waveform and underlying dynamics of atrial myocytes. The full base model formulation used here is found in the Supplementary Information. Unless otherwise specified, simulations characterizing the behavior of this model were performed at 1 Hz pacing frequency.

In order to represent the important differences between LA and PV myocytes, we have used published experimental data directly assessing differences in the membrane currents in myocytes isolated from both regions in the adult canine heart^22^. The only differences required to capture these effects in the LA and PV versions of the model are the maximum conductances of five transmembrane currents. Each has been shown to differ between LA and PV cells in^22^. The factors used to scale the conductance of the currents in the PV version of the model from the conductance of the currents in the LA version of the model are given in Table 1. The computational details of the human AP model used here are given in the Supplementary Information where the model also is compared to other AP models of the human atrial AP.

**Table 1:** Scaling factors for the currents of the pulmonary vein (PV) version of the base model, taken from^22^.

Parameter	PV scaling factor
$g_{\mathrm {K1}}$	0.58
$g_{\mathrm {Kr}}$	1.5
$g_{\mathrm {Ks}}$	1.6
$g_{\mathrm {to}}$	0.75
$g_{\mathrm {CaL}}$	0.70

### Modeling the AF-associated mutations

In this study, we have considered six different mutations that have been linked to AF^3^. Five of these mutations alter the function of specific ion channels in the sarcolemmal membrane of CMs and one affects the function of gap junction elements (connexins) that couple neighbouring CMs. We assume the mutations affect the function of individual channel proteins, and that the function of these proteins and functional effects of the mutations do not differ between LA and PV myocytes. Therefore, the effect of the mutations is modeled in the exactly same manner for the LA and PV cases. Below, we list the selected mutations and explain how each is represented in the models. This information is summarized in Table 2. For all the cases, the models for the wild type (WT) version of the channels are unchanged.

#### N588K

The N588K gain-of-function mutation in the KCNH2 gene encoding the ion channels responsible for the rapidly activating delayed rectifier $\hbox {K}^+$ current, $I_{\mathrm {Kr}}$, is associated with short QT syndrome type 1 and other cardiac arrhythmias, including AF^41,50^. McPate at al.^39^, have published detailed measurements of the difference between WT and N588K $I_{\mathrm {Kr}}$. We have fitted the $I_{\mathrm {Kr}}$ Markov model formulation from^51^ to these measurements. Specifically, the N588K mutation is represented by altering the transition rates between the inactivated and open states of the Markov model. The full formulation of the WT and N588K versions of the $I_{\mathrm {Kr}}$ model are specified in the Supplementary Information.

#### A545P

The A545P gain-of-function mutation occurs in the KCND3 gene encoding the Kv4.3 alpha subunit of channels carrying the transient outward $\hbox {K}^+$ current $I_{\mathrm {to}}$, and is associated with AF^42^. A545P mutant channels exhibit increased peak current, and slowed current inactivation. In accordance with measurements published by Olesen et al.^42^, we represent the mutation by increasing the maximum $I_{\mathrm {to}}$ conductance by 75% and the time constant of inactivation by 15%.

#### E299V

The E299V gain-of-function mutation in the KCNJ2 gene impacts ion channels responsible for the time-independent inwardly rectifying $\hbox {K}^+$ current, $I_{\mathrm {K1}}$. This mutation is associated with short QT syndrome type 3 and increased incidence of arrhythmias, including AF^43^. To represent the mutation in the models, we use the WT and E299V versions of the atrial $I_{\mathrm {K1}}$ formulations provided in^43^. These formulations are based on the Grandi et al. human atrial myocyte model^52^ and are fitted to WT and E299V $I_{\mathrm {K1}}$ measurements from^43^. The full formulation of the WT and E299V versions of the $I_{\mathrm {K1}}$ model are specified in the Supplementary Information.

#### E375X

The E375X mutation in the KCNA5 gene exerts loss-of-function effects in the Kv1.5 ion channels responsible for much of the measurable ultra-rapidly activating delayed rectifier $\hbox {K}^+$ current, $I_{\mathrm {Kur}}$, and has been causally linked to idiopathic lone AF^44^. Based on measurements of WT and E375X $I_{\mathrm {Kur}}$ from^44^, we represent this mutation in our model by reducing the maximum conductance of $I_{\mathrm {Kur}}$ to 10% of the WT value.

#### A130V

A130V is a loss-of-function mutation in SCN3B encoding the $\beta$3 subunit of the $\hbox {Na}^+$ channel complex that is responsible for $I_{\mathrm {Na}}$, and was uniquely identified in a patient with lone AF from a study of 477 AF patients of Han Chinese descent^45^. Based on measurements of WT and A130V $I_{\mathrm {Na}}$ from^45^, we represent this mutation in the model by reducing the maximum conductance of $I_{\mathrm {Na}}$ to 25% of the WT value.

#### c.932delC

The c.932delC mutation in the GJA1 gene encoding the gap junction protein connexin 43 (Cx43) has been identified as a genetic mosaicism underlying lone AF in a patient for whom cardiomyocyte-specific expression of this variant was responsible for the pathology^46^. Based on measurement of the gap junction conductance for WT and the c.932delC mutation from^46^, we represent this mutation in the associated model by reducing the gap junction conductance ($G_{\mathrm {gap}}$, see (2), below) to 25% of the WT value.

**Table 2:** Summary of how each of the selected mutations that have been linked to AF are represented in the LA and PV models. Here, GOF refers to gain-of-function mutations, and LOF refers to loss-of-function mutations. Complete mathematical specifications of each of these modified currents are given in the Supplementary Information.

Mutation	Effect	Modeling	Ref.
N588K	$I_{\mathrm {Kr}}$ GOF	Transition rates between the open and inactivated states adjusted	^39,41^
A545P	$I_{\mathrm {to}}$ GOF	$g_{\mathrm {to}}$ increased by 75% $\tau _{r_{\mathrm {to}}}$ increased by 15%	^42^
E299V	$I_{\mathrm {K1}}$ GOF	$I_{\mathrm {K1}}$ reformulated	^43^
E375X	$I_{\mathrm {Kur}}$ LOF	$g_{\mathrm {Kur}}$ reduced by 90%	^44^
A130V	$I_{\mathrm {Na}}$ LOF	$g_{\mathrm {Na}}$ reduced by 75%	^45^
c.932delC	$I_{\mathrm {gap}}$ LOF	$G_{\mathrm {gap}}$ reduced by 75%	^46^

### Finite element model of the PV ‘sleeve’

We perform finite element simulations of a defined portion of the ‘sleeve’ of the PV using a model, referred to as the EMI model, which represents the geometry and electric potentials of the extracellular space (E), the cell membrane (M) and the intracellular space (I) (see, e.g.,^28,29,31,32,53,54^). This model takes the form

1 $$\begin{aligned} \begin{aligned} \nabla \cdot \sigma _{i}\nabla u_{i}^{k}&=0&\mathrm {\;\;in\;}\Omega _{i}^{k},&\quad \; n_{e}\cdot \sigma _{e}\nabla u_{e} =-n_{i}^{k} \cdot \sigma _{i}\nabla u_{i}^{k} \equiv I_{m}^{k}&\mathrm {\;\;at\;}\Gamma _{k}, \\ \nabla \cdot \sigma _{e}\nabla u_{e}&=0&\mathrm {\;\;in\;} \Omega _{e},&\qquad v_{t}^{k} =\tfrac{1}{C_{m}}(I_{m}^{k}-I_{\mathrm {ion}}^{k})&\mathrm {\;\;at\;}\Gamma _{k},\\ u_{e}&= 0&\mathrm {\;\;at\;}\partial \Omega _{e}^{D},&\qquad u_{i}^{k}-u_{i}^{{\tilde{k}}} =w^k&\mathrm {\;\;at\;}\Gamma _{k,{\tilde{k}}}, \\ n_{e} \cdot \sigma _{e}\nabla u_{e}&= 0&\mathrm {\;\;at\;}\partial \Omega _{e}^{N},&\qquad n_{i}^{{\tilde{k}}}\cdot \sigma _{i}\nabla u_{i}^{{\tilde{k}}} =-n_{i}^{k}\cdot \sigma _{i}\nabla u_{i}^{k}\equiv I_{k,{\tilde{k}}}&\mathrm {\;\;at\;}\Gamma _{k,{\tilde{k}}},\\ u_{i}^{k}-u_{e}&=v^{k}&\mathrm {\;\;at\;}\Gamma _{k},&\qquad w^k_{t} =\tfrac{1}{C_{g}}(I_{k,{\tilde{k}}}-I_{\mathrm {gap}}^k)&\mathrm {\;\;at\;}\Gamma _{k,{\tilde{k}}}, \\ s^{k}_{t}&= F^k&\mathrm {\;\;at\;}\Gamma _k, \end{aligned} \end{aligned}$$

for all myocytes *k* and neighboring myocytes ${\tilde{k}}$^31^. Here, $u_e$ (in mV) is the electric potential in the extracellular space, $\Omega _e$, and $u_i^k$ (in mV) is the electric potential in the intracellular space of myocyte *k*, $\Omega _i^k$. Moreover, $v^k=u_i^k-u_e$ (in mV) is the transmembrane potential of myocyte *k*, defined at the membrane $\Gamma _k = \Omega _e \cap \Omega _i^k$, and $w^k=u_i^k-u_i^{{\tilde{k}}}$ (in mV) is the potential difference between myocyte *k* and its neighboring myocyte ${\tilde{k}}$, defined at the intercalated disc $\Gamma _{k,{\tilde{k}}} = \Omega _i^k \cap \Omega _i^{{\tilde{k}}}$. Furthermore, $\sigma _i$ and $\sigma _e$ (in mS/cm) is the conductivity of the extracellular and intracellular spaces, respectively, and $C_m$ and $C_g$ (in $\mu$F/$\hbox {cm}^2$) is the specific capacitance of the cell membrane and the intercalated discs, respectively. In addition, $I_{\mathrm {ion}}^k$ (in $\mu$A/$\hbox {cm}^2$) represents the sum of ionic currents through ion channels, pumps and exchangers expressed in the sarcolemma, represented by the base model described in the “Membrane models for PV and LA cardiomyocytes” section. The state variables of the base model are referred to as $s^k$, and their dynamics are referred to as $F^k$. Note that, $I_{\mathrm {gap}}^k$ (in $\mu$A/$\hbox {cm}^2$) is the current density between neighbouring myocytes, specified by the passive model

2 $$\begin{aligned} I_{\mathrm {gap}}^k = \frac{1}{R_{\mathrm {gap}}}w^k = G_{\mathrm {gap}}w^k, \end{aligned}$$

where $G_{\mathrm {gap}}$ (in mS/$\hbox {cm}^2$) is the conductance of the gap junctions and $R_{\mathrm {gap}}$ (in k$\Omega$ $\hbox {cm}^2$) is the corresponding resistance of the gap junctions. The parameters used in the EMI model simulations are specified in Table 3, and the EMI model equations are solved numerically using an MFEM^55,56^ finite element implementation of the operator splitting algorithm introduced in^30,57^. A 500 ms simulation of cell to cell conduction within our PV sleeve substrate required a simulation time of approximately 8 days (190 hours).

**Table 3:** Default parameter values used in the EMI model simulations, see, e.g.,^30^.

Parameter	Value
$C_m$	$1\;\mu \mathrm {F/cm}^2$
$C_{g}$	$0.5\;\mu \mathrm {F/cm}^2$
$\sigma _i$	$4\;\mathrm {mS/cm}$
$\sigma _e$	$20\;\mathrm {mS/cm}$
$R_{\mathrm {gap}}^*$ (longitudinal)	0.0005 k$\Omega$ $\hbox {cm}^2$
$R_{\mathrm {gap}}^*$ (transverse)	0.001 k$\Omega$ $\hbox {cm}^2$
$\Delta t$	0.001 ms
$M_{\mathrm {it}}$, $N_{\mathrm {it}}$	1

#### PV sleeve geometry

**Figure 1:**
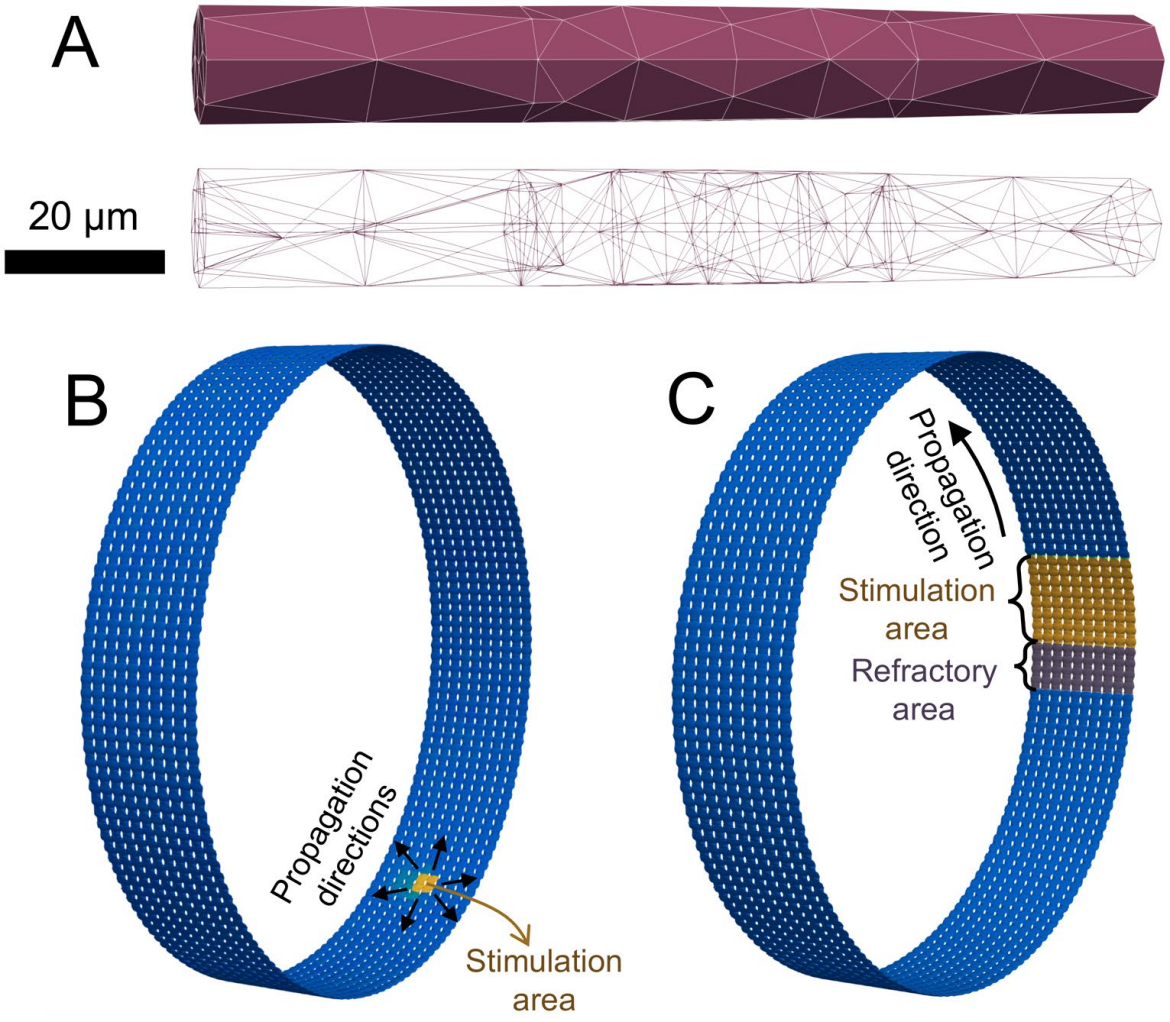
Single myocyte and PV sleeve geometry used in these EMI model simulations. A: The upper illustration shows the surface of a single myocyte within the PV sleeve, and the lower illustration shows the full finite element mesh of the myocyte. In the mesh, each cell is represented by about 85 nodes, of which approximately 80 are located on the membrane or at the intercalated discs. B and C: Illustration of a cylinder of myocytes making up the PV sleeve. Each cell is shaped as illustrated in Panel A. The figure in Panel B also illustrates the stimulation protocol used in the simulations performed to investigate excitability, and the figure in Panel C illustrates the stimulation protocol used to investigate re-entry. Note that to improve the visibility, the cylinders in Panels B and C are not shown to scale.

We represent essential features of the geometry of the PV sleeve by constructing a collection of coupled myocytes that form a cylinder having a diameter of 1.5 cm (similar to the diameter of a human PV^58^). Each myocyte is shaped as a cylinder with a diameter varying from 13 $\mu$m at the cell ends to 14 $\mu$m at the cell center, and each myocyte is about 120 $\mu$m long^59^. In our formulation, the cylinder of myocytes consists of 393 cells placed longitudinally around the cylinder with 10 rows of cells positioned along the cylinder. The computational mesh of a single cell is illustrated in Fig. 1A and an associated cylinder of cells is illustrated in Fig. 1B–C.

#### Distribution of cell properties

We assume that the properties of the CMs forming the sleeve of the PV varies between those of PV and LA CMs but that there is no mean gradient from one end of the cylinder to the other. This arrangement is intended to specifically represent the subsection of the sleeve that is at the border between the vein and remote atrial myocardium. We represent this by drawing a number $\alpha$ that is 0 or 1 for each of the 393$\times$10 myocytes and letting the conductance of the five currents known to be different between PV and LA CMs (see Table 1) for each cell be given by

3 $$\begin{aligned} g_{\mathrm {cell}} = \alpha \cdot g_{\mathrm {LA}} + (1-\alpha )\cdot g_{\mathrm {PV}}, \end{aligned}$$

where $g_{\mathrm {LA}}$ and $g_{\mathrm {PV}}$ are the LA and PV conductance values, respectively.

Somewhat similarly, the complex localized fibre arrangements and slow and complex conduction that have been observed in the PV sleeve^20^ are represented by scaling the gap junction resistance between default values and values corresponding to a considerably reduced cell coupling. This is done by drawing random numbers $\beta$ between 0 and 1 for each intercalated disc and letting

4 $$\begin{aligned} R_{\mathrm {gap}} = \beta \cdot R_{\mathrm {gap}}^{*} + (1-\beta )\cdot 30R_{\mathrm {gap}}^*, \end{aligned}$$

where $R_{\mathrm {gap}}^{*}$ are the default gap junction resistances reported in Table 3.

The numbers $\alpha$ and $\beta$ are drawn randomly *once* from a uniform distribution and the same values are reused in all simulations.

### Definition of excitability and re-entry

We have performed EMI model simulations based on the PV sleeve to investigate how the selected ion channel or connexin mutations may affect the excitability and/or potential for re-entry in the tissue. Below, we describe the protocols used in these simulations.

#### Excitability

In order to investigate the excitability of the PV sleeve, we apply a 2 ms rectangular stimulus waveform ($I_{\mathrm {stim}}$) to 2$\times$2 myocytes (see Fig. 1B). We evaluate how intense this depolarizing stimulus current must be to initiate an AP in the tissue, where a larger required $I_{\mathrm {stim}}$ denotes reduced tissue excitability. We define excitability, *E*, as

5 $$\begin{aligned} E(\mathrm {MT}) = \frac{I_{\mathrm {stim}}^{\mathrm {WT}}}{I_{\mathrm {stim}}^{\mathrm {MT}}}, \end{aligned}$$

where $I_{\mathrm {stim}}^{\mathrm {WT}}$ (in A/F) is the required stimulation current in the WT case and $I_{\mathrm {stim}}^{\mathrm {MT}}$ (in A/F) is the required current for the mutation under consideration. Here, *required* refers to the smallest $I_{\mathrm {stim}}$ capable of initiating an AP.

#### Re-entry

In order to investigate how each selected mutation affects the likelihood of sustaining re-entry, we performed EMI model simulations in which 8 columns of myocytes around the cylinder are stimulated by setting their initial membrane potential to $v=-10$ mV. In addition, we enforce the condition $\frac{\partial v}{\partial t}=0$ in 4 columns of myocytes on one side of these 8 columns of myocytes, representing a refractory area. This condition is enforced until the propagating AP approaches these myocytes after traveling once around the cylinder. The approaching AP is monitored in each simulation, so that the point in time when the condition ends is adjusted for each individual simulation. We run the simulation for 500 ms and then determine whether the membrane potential of any of the myocytes is more depolarized than $-65$ mV anywhere in the cylinder at the end of the simulation. If so, we say that re-entry is obtained in the PV sleeve. The setup used to initiate re-entry is illustrated in Fig. 1C.

### Defining biomarkers

In the results reported below, we utilize a number of different biomarkers, each computed from the solution of the ordinary differential equations defining the membrane models for LA and PV myocytes and from the EMI model simulations of the PV sleeve with a mix of LA and PV myocytes. In this section, we describe definitions of these biomarkers.

#### Electrophysiological

*(a) Resting membrane potential (RMP)* The resting membrane potential (RMP) is defined as the membrane potential obtained 10 ms before the stimulation current that initiates the action potentials is applied.

*(b) Action potential amplitude (APA)* The action potential amplitude (APA) is defined as the difference between the RMP and the maximum value of the membrane potential obtained during an action potential.

*(c) Maximal upstroke velocity* ($\hbox {dvdt}_{\max }$) We define the maximal upstroke velocity ($\hbox {dvdt}_{\max }$) as the maximum value of the first derivative of the membrane potential with respect to time during the upstroke of the action potential.

*(d) Action potential duration (APD)* We define the action potential duration at *p* percent repolarization (e.g., APD50, APD80, APD90) as the time between the time when $\hbox {dvdt}_{\max }$ is reached and the time during repolarization when membrane potential reaches a value below $v_{\min }+\left( 1-\frac{p}{100}\right) (v_{\max }-v_{\min })$, where $v_{\min }$ is the minimum value of the membrane potential obtained between two action potentials and $v_{\max }$ is the maximum value of the membrane potential during an action potential.

#### [$\hbox {Ca}^{2+}]_i$ dynamics

*(a)* $\hbox {Ca}^{2+}$ *transient amplitude (CaA)* We define the [$\hbox {Ca}^{2+}$] transient amplitude (CaA) as the difference between the maximum value of the cytosolic $\hbox {Ca}^{2+}$ concentration obtained during an action potential and the lowest value obtained between two action potentials.

*(b)* [$\hbox {Ca}^{2+}$] *transient duration (CaD)* The $\hbox {Ca}^{2+}$ transient duration at *p* percent (e.g., CaD50, CaD90) is defined in the same manner as the APD.

#### Tissue dynamics

*(a) Conduction velocity (CV)* We compute the conduction velocity in the EMI model simulations used to investigate re-entry by recording the times (after stimulation) at which the myocyte in row 5, column 50 ($t_1$) and the myocyte in row 5, column 100 ($t_2$) each first reach positive membrane potentials. The conduction velocity is then computed as

6 $$\begin{aligned} \text {CV} = \frac{d}{t_2-t_1}, \end{aligned}$$

where $d = 50\cdot 120\;\mu$m = 0.6 cm is the distance between the center of the two myocytes. This corresponds to the longitudinal conduction velocity of the cells, in the direction around the cylinder.

*(b) Wavelength,* $\lambda$ For the excitation wave traveling around the cylinder shown in Figure 1, we also estimate the length around the cylinder that is covered by the wave. This conduction wavelength, $\lambda$, is computed as

7 $$\begin{aligned} \lambda = \text {CV}\cdot \text {APD90}. \end{aligned}$$

## Results

In this section, we present results from our simulations investigating the effects of selected ion channel protein mutations on the electrophysiological properties of a simulated functional sleeve of the PV. We first present key properties of the wild type models based mainly on the membrane currents of LA and PV myocytes, and we then examine how these properties are affected by each of the six mutations. Next, we investigate the effect of the mutations in EMI model simulations of the multicellular PV sleeve tissue. Specifically, we investigate whether the excitability of the CMs in the sleeve is altered and whether the mutations change the electrophysiological properties of the sleeve in a manner such that a re-entrant wave is sustained.

### Properties of membrane models for LA and PV

In order to accurately represent the membrane currents and intracellular $\hbox {Ca}^{2+}$ fluxes of both LA and PV human myocytes and relate our findings to previous publications, we use a modified version of the base model from^40,47^. The differences between the LA and PV versions of our model are closely based on measured differences in transmembrane current densities from^22^. These are specified in Table 1.

**Figure 2:**
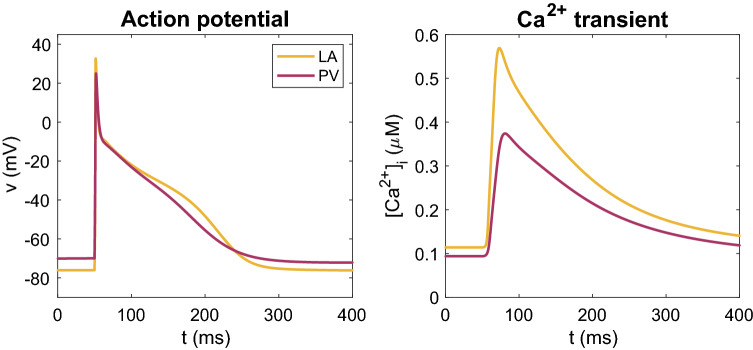
Action potentials (left) and [$\hbox {Ca}^{2+}$] transients (right) for the LA and PV versions of our base model obtained at steady-state in response to a 1 Hz stimulus train.

**Table 4:** Biomarker values for the LA and PV versions of our base model at 1 Hz pacing. Selected biomarkers include the resting membrane potential (RMP), the action potential amplitude (APA), the maximal upstroke velocity of the action potential ($\hbox {dvdt}_{\max }$), the action potential durations at 90% and 50% repolarization (APD50 and APD90), the amplitude of the cytosolic $\hbox {Ca}^{2+}$ transient (CaA), and the cytosolic $\hbox {Ca}^{2+}$ transient durations at 90 and 50% (CaD50 and CaD90). These biomarkers are defined in the “Defining biomarkers” section.

	LA	PV
RMP	$-76$ mV	$-70$ mV
APA	109 mV	95 mV
$\hbox {dvdt}_{\max }$	198 V/s	101 V/s
APD90	187 ms	172 ms
APD50	48 ms	51 ms
CaA	0.45 $\mu$M	0.28 $\mu$M
CaD90	277 ms	323 ms
CaD50	97 ms	120 ms

**Figure 3:**
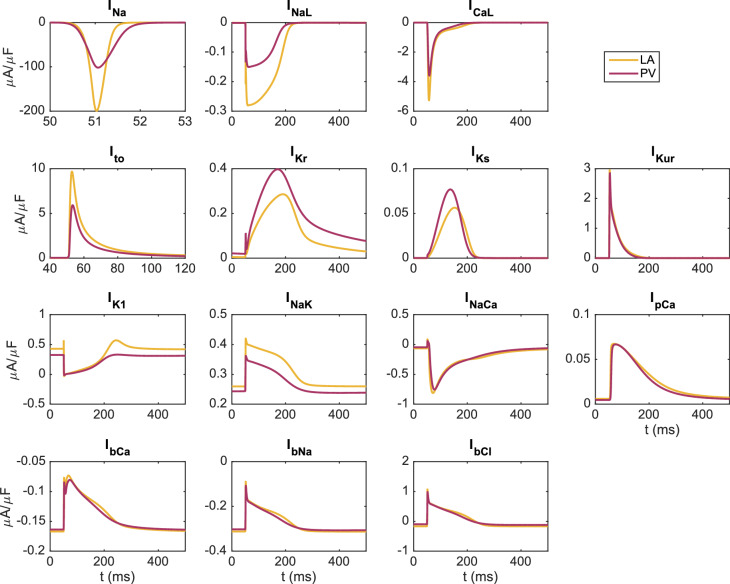
Membrane currents during an action potential in the LA and PV versions of the base model. Definitions and mathematical specifications for each of these currents are provided in the Supplementary Information.

**Figure 4:**
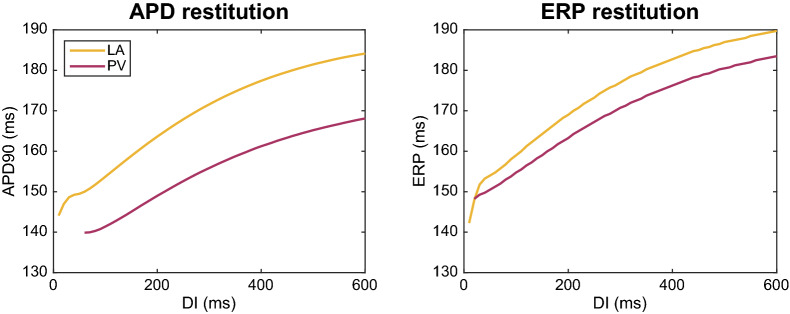
Action potential duration (APD) and effective refractory period (ERP) restitution curves for the LA and PV base models. For the APD restitution curve, the APD90 value is computed for a number of different diastolic interval (DI) values. The diastolic interval is defined as the time between the time point when the membrane potential has returned to 90% repolarization in the previous action potential (resulting from an S1 stimulation) to the time the next stimulation (S2) is applied. To compute the ERP restitution, a third stimulation (S3) is applied for each S2 DI. This S3 stimulation (lasting for 3 ms and of an amplitude about twice as large as was needed to generate an action potential from rest) is applied at progressively shorter time intervals after the S2 stimulation and the ERP is defined as the longest S2S3 interval that failed to capture^60^.

Figure 2 shows the AP and cytosolic [$\hbox {Ca}^{2+}$] transient for the LA and PV versions of the base model. In Table 4, we compare a number of biomarkers for the two versions of the model. It is apparent that both human base models capture observed semi-quantitative differences between canine LA and PV myocytes from^22^. Specifically, the resting membrane potential is depolarized for the PV model compared to the LA model, and the maximum upstroke velocity of the action potential (AP) is considerably lower for PV than LA. In addition, the APD is longer for LA myocytes than for PV myocytes. All of these characteristics are consistent with the experimental observations^22^. Figure 3 shows the current densities for the different membrane currents of the LA and PV versions of the base model during an action potential. In Fig.  4, we also present the dynamic restitution properties of both models, which are key determinants of arrhythmia potential. The baseline difference in APD between the PV and LA models is retained across diastolic intervals (DI’s), resulting in very similar restitution slopes, both of which are well below 1 until the DI approaches zero. Therefore, these restitution properties are unlikely to promote arrhythmogenic mechanisms involving APD alternans in either model. The effective refractory periods (ERPs) are more similar for the two models with the PV model still exhibiting a slight reduction. Again, the ERP restitution slopes are much less than unity until DI approaches only 10-20 ms (after 90% replarization) for the LA model.

### Effect of the mutations on the membrane models

**Figure 5:**
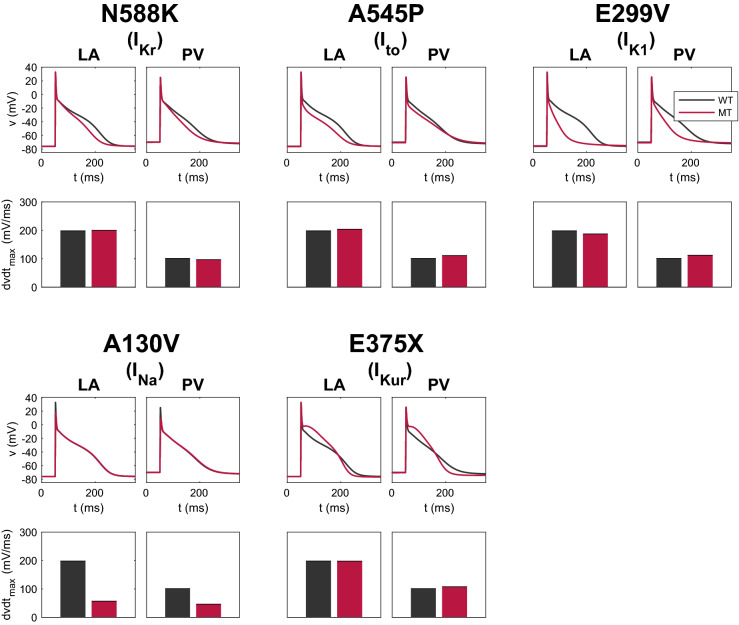
Effects of selected mutations on the AP and maximal upstroke velocity in single myocyte simulations of the LA and PV versions of our membrane model. The black lines and bars refer to WT, and the red lines and bars refer to the mutant cases. Note that the c.932delC mutation (in connexin 43) is not included here because the c.932delC mutation does not affect any of the parameters of the membrane models. However, it can have a significant effect on intercellular coupling, CV and arrhythmogenic substrate in the multicellular (syncytium) simulations.

In Fig. 5 and Table 5, we investigate the effect of incorporating different mutations in the LA and PV versions of the base model as described in the “Modeling the AF-associated mutations” section. Note that a number of mutations (N588K, A545P and E299V) lead to a shortened APD for both PV and LA cells, and that the degree of shortening varies among the mutations. For the A130V mutation, we observe a significant decrease in the maximal upstroke velocity. For the E375X mutation, early phase II repolarization is markedly slowed, as is to be expected for this major loss-of-function $I_{\mathrm {Kur}}$ mutant, although terminal repolarization and the indices of later APD are accelerated. Note that even though the E299V mutation affects the $I_{\mathrm {K1}}$ current, which is one of the most prominent currents during rest (see Fig. S3 in the Supplementary Information), the resting membrane potential of the LA and PV models appears to be relatively unaltered by this mutation (see Table 5). This may be explained by the fact that the difference between the WT and E299V $I_{\mathrm {K1}}$ is quite small for values of the membrane potential close to the resting membrane potential of the LA and PV models and more prominent for less depolarized values of the membrane potential (see Fig. S4 in the Supplementary Information). Since the c.932delC mutation affects only gap junction function, which is only implemented for the tissue simulations, none of the parameters of the membrane model are changed for this mutation.

**Table 5:** Comparisons between computed WT and mutant biomarker values for simulations of the LA and PV versions of the membrane model driven at 1 Hz. These comparisons are based on changes in the same biomarkers as in Table 4, which are defined in the “Defining biomarkers” section. The numbers in parentheses report the deviation from WT. Note that the c.932delC mutation (in connexin 43) is not included here because these biomarkers are computed from simulations of the single myocyte LA and PV membrane models, and the c.932delC mutation does not affect any of the parameters of the membrane models.

	RMP (mV)		APA (mV)		$\hbox {dvdt}_{\mathrm {max}}$ (V/s)		APD90 (ms)		APD50 (ms)		CaA ($\mu$M)		CaD90 (ms)		CaD50 (ms)	
**LA**																
WT	$-76$		109		198		187		48		0.45		277		97	
N588K ($I_{\mathrm {Kr}}$)	$-76$	($-0$%)	109	($+0$%)	200	($+1$%)	155	($-17$%)	41	($-16$%)	0.39	($-13$%)	289	($+4$%)	104	($+7$%)
A545P ($I_{\mathrm {to}}$)	$-76$	($-0$%)	109	($+0$%)	204	($+3$%)	158	($-15$%)	17	($-64$%)	0.36	($-20$%)	295	($+6$%)	100	($+3$%)
E299V ($I_{\mathrm {K1}}$)	$-75$	($+1$%)	107	($-2$%)	187	($-6$%)	83	($-56$%)	16	($-67$%)	0.29	($-37$%)	321	($+16$%)	104	($+7$%)
A130V ($I_{\mathrm {Na}}$)	$-76$	($+0$%)	94	($-14$%)	57	($-71$%)	192	($+2$%)	81	($+66$%)	0.44	($-3$%)	282	($+2$%)	100	($+2$%)
E375X ($I_{\mathrm {Kur}}$)	$-76$	($+0$%)	109	($-0$%)	198	($-0$%)	171	($-9$%)	88	($+82$%)	0.45	($-1$%)	274	($-1$%)	96	($-2$%)
**PV**																
WT	$-70$		95		101		172		51		0.28		323		120	
N588K ($I_{\mathrm {Kr}}$)	$-70$	($+0$%)	94	($-1$%)	97	($-4$%)	144	($-17$%)	40	($-21$%)	0.25	($-10$%)	334	($+3$%)	122	($+2$%)
A545P ($I_{\mathrm {to}}$)	$-71$	($-1$%)	96	($+1$%)	111	($+10$%)	173	($+0$%)	27	($-46$%)	0.27	($-4$%)	326	($+1$%)	116	($-4$%)
E299V ($I_{\mathrm {K1}}$)	$-71$	($-1$%)	97	($+2$%)	112	($+11$%)	106	($-39$%)	22	($-56$%)	0.23	($-18$%)	345	($+7$%)	119	($-1$%)
A130V ($I_{\mathrm {Na}}$)	$-70$	($+0$%)	80	($-16$%)	46	($-54$%)	182	($+5$%)	78	($+54$%)	0.26	($-7$%)	331	($+2$%)	125	($+4$%)
E375X ($I_{\mathrm {Kur}}$)	$-70$	($-1$%)	96	($+1$%)	108	($+7$%)	146	($-15$%)	78	($+55$%)	0.25	($-12$%)	333	($+3$%)	133	($+11$%)

### Effect of the mutations on excitability

**Figure 6:**
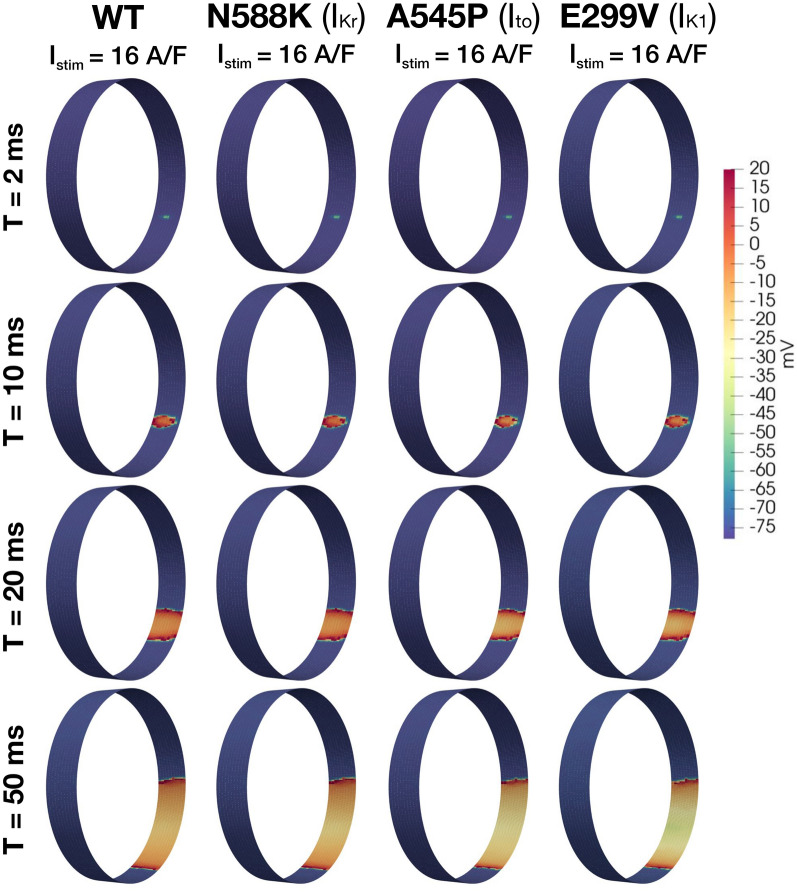
Excitability analysis for WT, and for the N588K, A545P, and E299V mutations. Intracellular potential of myocytes in the PV sleeve is illustrated at four different times (rows) for each mutation (columns). In all cases a regenerative AP was generated and propagated around the tissue cylinder. The stimulus intensity (applied to 2$\times$2 cells for 2 ms) required to initiate the propagating action potential in each case is provided in the title for each mutation. Note that to improve the visibility, the transversal axis of the myocytes has been stretched by a factor of 20.

**Figure 7:**
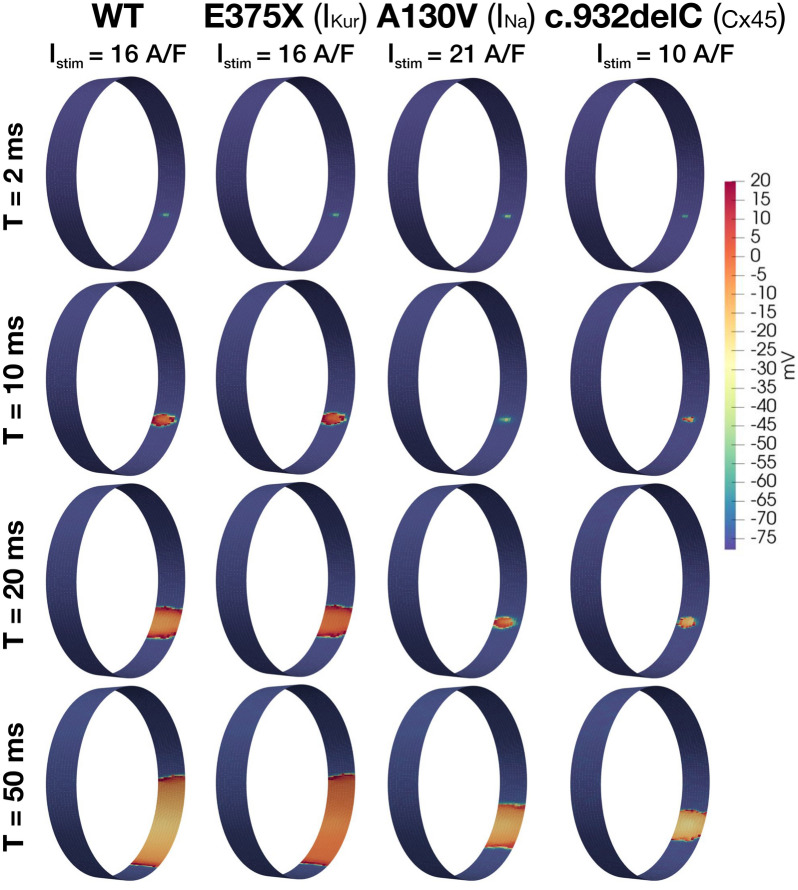
Excitability analysis for WT, and for the E375X, A130V, and c.932delC mutations. This presentation format is the same as that of Fig. 6.

The effect of each selected mutation on the excitability of the myocytes in the PV sleeve has been investigated using the protocol described in the “Excitability” section. Figures 6 and 7 show the intracellular potential in these simulations at pre-determined, fixed points in time. In Fig. 6, we observe that a 2 ms stimulus current of 16 A/F is needed to initiate an AP in the WT tissue cylinder, and that this minimum stimulus intensity is unchanged for the N588K, A545P, E299V and E375X mutants. These findings are largely to be expected given that these mutations all impact $\hbox {K}^+$ currents, but did not influence resting membrane potential.

In Fig. 7, we observe that for the c.932delC mutation, a smaller stimulus current is sufficient to generate a propagating AP, indicating an increased excitability for this mutation. On the other hand, a stronger stimulus is required for the A130V mutation, thus indicating decreased excitability.

### Effect of the mutations on re-entry

**Figure 8:**
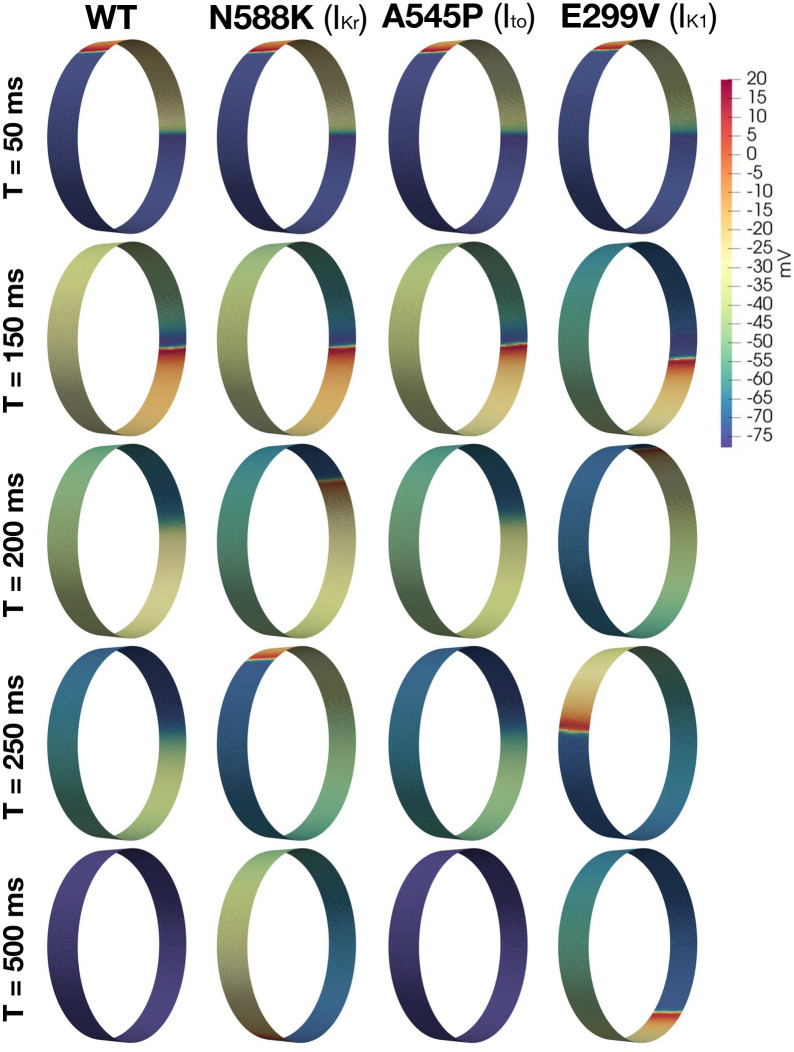
EMI-based multicellular simulations of electrical propagation and re-entry in the cylinder segment of the PV sleeve tissue. We use the simulation protocol described in the “Re-entry” section for investigating re-entry. Data are illustrated for WT, and the N588K, A545P, and E299V mutations (columns). Specifically, we show the intracellular potential of the cells in the PV sleeve at five different times after stimulation (rows). Note that to improve the visual resolution, the transversal axis of the myocytes has again been stretched by a factor of 20.

**Figure 9:**
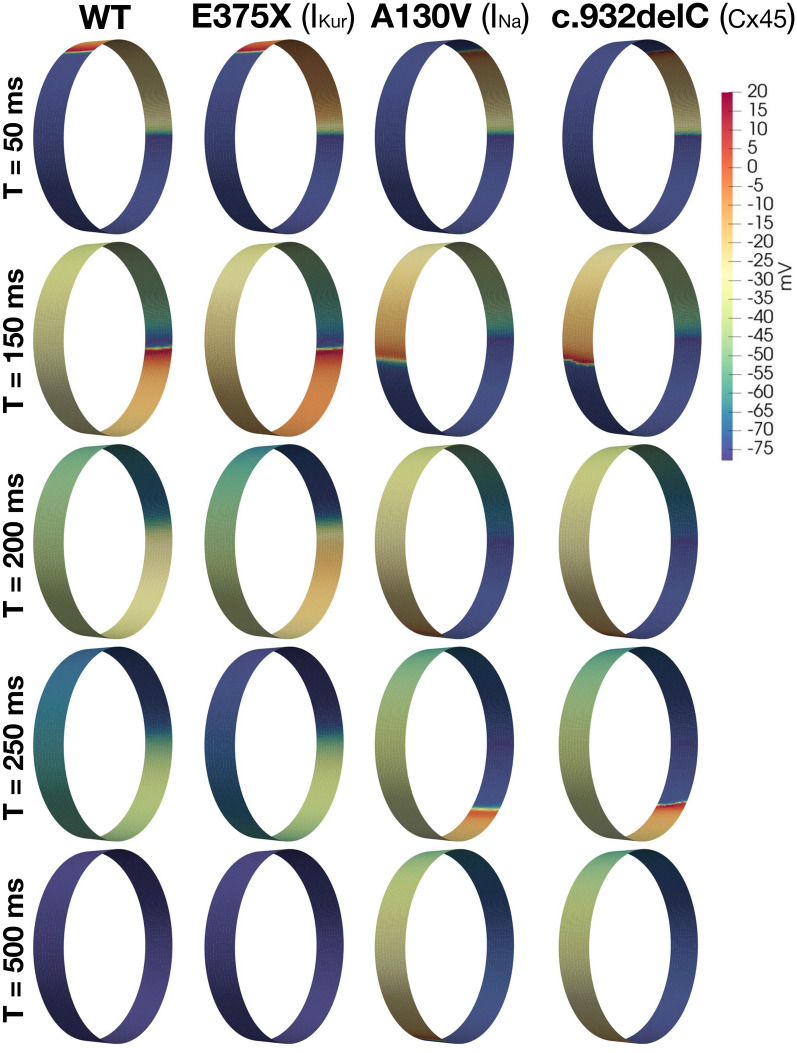
EMI-based multicellular simulations for WT, and for the E375X, A130V, and c.932delC mutations. The format of the figure is analogous to that of Fig. 8.

To investigate how the same mutations affect the initiation and/or maintenance of re-entry, we use the protocols described in the “Re-entry” section. Figures 8 and 9 show the intracellular potential of these simulations at some different points in time. In Figs. 8 and  9, we observe that in the WT case, a propagating wave begins to travel around the tissue cylinder, but upon reaching the stimulation site it encounters a refractory region and terminates. Thus, re-entry is prevented. The same mechanism is observed for the A545P and E375X mutations.

In contrast, the shorter APs and refractory periods for the N588K and E299V mutations (Fig. 5), permit the approaching wavefront to re-excite this region. Consequently, the propagating wave continues to travel around the cylinder as a re-entrant wave.

For the A130V and c.932delC mutations, APD is not changed considerably (Fig. 5), but for A130V the maximal upstroke velocity is significantly decreased and for the c.932delC mutation, gap junction conductance is reduced by 75%. As a result, CV is considerably slower for both cases, thus permitting the myocytes at the stimulation site to fully repolarize, recover from refractoriness, and re-excite upon arrival of the propagating AP.

#### The wavelength of the conducted impulse

The wavelength of the conducted impulse is usually defined as the conduction velocity times the refractory period, see, e.g.,^1^, and a re-entry wave is expected to be sustained if the geometric conduction pathway is larger than the wavelength. In our case, we expect re-entry to be possible if the wavelength is shorter that the circumference of the circle of myocytes illustrated in Fig. 1. In computations, the APD is often used as a proxy for the refractory period. In Table 6, we report the wavelength in our EMI model simulations, as defined by (7), in addition to the action potential duration (APD90), the conduction velocity (CV), the excitability (defined in the “Excitability” section) and the re-entry (as defined in the “Re-entry” section). In all but one case, we observe re-entry only if the wavelength is shorter than the circumference of the PV sleeve substrate. However, for the N588K mutation, we note that even though the wavelength is 4.9 cm and the circumference of the PV sleeve is 4.7 cm, re-entry is still observed. This is most likely because the APD90 value used in the calculation of the wavelength is only a rough approximation of the refractory period, and is discussed further in the Supplementary Information.

**Table 6:** Summary of the results of the EMI model simulations of the PV sleeve for WT and six mutations linked to AF. Definitions of the properties are given in the “Definition of excitability and re-entry” and “Defining biomarkers” sections, and the numbers in parentheses report the deviation from WT. The distance around the PV sleeve cylinder (of radius 1.5 cm) is approximately 4.7 cm.

Mutation	APD90 (ms)		CV (cm/s)		$\lambda$ (cm)		Excitability	Reentry
WT	190		30.5		5.8		1.00	N
N588K ($I_{\mathrm {Kr}}$)	160	($-16\%$)	30.6	($+0\%$)	4.9	($-16\%$)	1.00	Y
A545P ($I_{\mathrm {to}}$)	184	($-3\%$)	30.6	($+0\%$)	5.6	($-2\%$)	1.00	N
E299V ($I_{\mathrm {K1}}$)	110	($-42\%$)	29.9	($-2\%$)	3.3	($-43\%$)	1.00	Y
E375X ($I_{\mathrm {Kur}}$)	160	($-16\%$)	30.5	($-0\%$)	4.9	($-16\%$)	1.00	N
A130V ($I_{\mathrm {Na}}$)	202	($+7\%$)	15.9	($-48\%$)	3.2	($-44\%$)	0.76	Y
c.932delC (Cx43)	186	($-2\%$)	16.2	($-47\%$)	3.0	($-48\%$)	1.60	Y

### Summary of results

To assess the ability of our EMI framework to distinguish mechanisms of arrhythmia in the PV sleeve we have imposed the effects of a range of arrhythmia-associated mutations on a schematic PV geometry incorporating heterogeneous intercellular coupling and cell electrophysiology at single myocyte resolution. The results of these simulations are, as mentioned above, summarized in Table 6. The simulations reveal that the dominant modes of arrhythmogenesis for these mutants are qualitatively consistent with their effects on single myocyte electrophysiology, but that they differ in tissue arrhythmogenic potential due to their severity or modulation of tissue electrotonic properties. To be specific, mutations that either considerably reduce APD (and refractory period, i.e. N588K and E299V), or slow conduction velocity (A130V, c.932delC) induce “leading circle” type reentry in our small and heterogeneous tissue geometry. Other mutations that cause less severe AP abbreviation, namely A545P and E375X, are unable to support genuine reentrant conduction in PV simulations, and thus are likely to require combination with additional AP shortening stimuli *in* *vivo*.

The two mutations that effect linear conduction velocity (A130V and c.932delC), also impacted excitability in the PV tissue by modulating its electrotonic properties. A130V reduced excitability by impairing the inward $\hbox {Na}^+$ current response to the trigger stimulus, while c.932delC enhanced excitability by reducing the electrotonic load imposed by the surrounding tissue.

## Discussion

The borders between the PV sleeves and working atrial myocardium are small regions of tissue exhibiting pronounced electrophysiologic heterogeneity and structural complexity. Here we have asked whether a recently developed mathematical model (EMI), which is specifically built to interrogate heterogeneities at the level of single cardiac myocytes, is capable of identifying clinically meaningful arrhythmogenic tendencies in this region. We specifically selected a range of genetic mutations known to contribute significantly to the genetic basis of either lone AF, or AF with only comorbid arrhythmias (e.g., short QT syndrome). We then assessed whether the arrhythmogenic impacts of these mutants could be identified by an EMI implementation of a thin cross-section of the PV-LA border. Our model incorporates heterogeneity of myocyte electrophysiology as defined by patch-clamp measurements in isolated PV and LA myocytes, and sharp microelectrode tissue recordings. This approach was readily capable of assessing the proarrhythmic influence of these mutations, and also of revealing one dominant mode of arrhythmogenesis for each. Specifically, it was able to identify and begin to characterize the propensity for re-entrant behavior among mutants that expand the region of excitable tissue (in this small geometry), either by reducing conduction velocity or abbreviating the tissue APD (and hence the tissue refractory period). This computational approach was also able to distinguish the propensity for two mutations impacting $I_{\mathrm {Na}}$ (A130V) and Cx43 (c.932delC) to markedly alter tissue excitability; and as expected, to do so in opposite directions. These results suggest that this type of EMI implementation may provide a viable platform for discriminating other clinically important pro- and anti-arrhythmic influences. Most notably for candidate pharmaceutical treatments specific to PV arrhythmogenesis.

### The role of high-resolution modeling for understanding arrhythmogenesis in the PV sleeves

Since the initial identification of the PV-LA junctions as the primary sites of AF initiation^18^, much progress has been made towards understanding the unique characteristics of this region that so strongly predispose it to generating abberant and disruptive electrical activity. Some of these factors are structural, and include the geometry of the tissue (a long, thin cylinder of myocardium directly bordered by the insulating fibrous veinous wall), sharp discontinuities in myocyte fiber direction at the PV-LA border^61,62^, and characteristic fibrous invasions that can create conduction discontinuties and promote local circuit formation^62–64^. Other important differences are electrophysiologic. PV myocytes have the shortest APs of any myocyte subtype in the human heart^65^, and exhibit a clear propensity for triggered activity^66,67^. Some have suggested that a specific population of myogenic or spontaneously active myocytes exist in the PV sleeves^68^, although this has not been broadly confirmed.

A range of computational studies have incorporated some of these characteristics and begun to create a biophysically defined heirarchy of arrhythmogenic mechanisms in the PV sleeves. Earlier work from Cherry et al.^69^ clearly demonstrated an important interaction of heterogeneity in myocyte-to-myocyte coupling with tissue size for promoting re-entry in a pseudo-PV sleeve. More recent computational efforts have sought to apply clinical imaging data to understand how macroscopic properties of PV electrophysiology and structure interact with electrical activity in the atrial free wall^12,16,70–74^. These approaches often incorporate measured variations in fibre direction, together with electrophysiologic heterogeneities in regions defined at the centimeter scale, in geometries of individual patient atria^12,16,72,73^. Some have also included heuristically defined fibrotic regions within the PV sleeve^12^. Together, these studies have suggested a range of structural and dynamic substrate components are important for permitting macroscopic arrhythmia to initiate (or anchor) around the PV-LA junction, and then propagate into the atrial myocardium. However, because all of these studies employed the bidomain or monodomain models they were not able to address electrophysiologic heterogeneities or structural characteristics smaller than a length scale of several millimetres. This precludes investigating a range of properties that rely on local heterogeneities or microscopic fibrotic barriers to create micro-reentrant circuits, or spontaneous foci at the PV-LA border. These proarrhythmic events have been suggested by many studies, and the PV-LA border is among the most probable locations for these proarrhythmic mechanisms in humans^62–64^.

### Simulated pro-arrhythmic properties of AF mutations at the PV-LA junction

While the mutations we have studied here were not specifically chosen for their link to PV sleeve arrhythmias, all are channelopathies that have been causally linked to lone AF or AF that is only comorbid with other arrhythmia disorders. As such they are electrophysiologically well-characterized and known to predispose to AF without a requirement for broader confounding comorbidities (e.g. heart failure). Our implementations of these mutations faithfully recapitulated their electrophysiologic phenotypes at the level of single myocytes. Some of these were quite subtle (A545P, $I_{\mathrm {to}}$), while others were much more severe (E299V, $I_{\mathrm {K1}}$). Our EMI tissue framework was able to distinguish an arrhythmic propensity in 4 of the 6 mutants, and it was only the the E375X ($I_{\mathrm {Kur}}$) and A545P ($I_{\mathrm {to}}$) mutants that did not exhibit clear arrhythmic activity. In all cases this arrhythmic activity was re-entrant, that is, we did not observe spontaneous activity in any of the tissue simulations. However, this likely reflects greater sensitivity of our re-entry protocol, compared to our excitability protocol, for distinguishing arrhythmic potential. In particular, we did not incorporate any instances of the PV or LA myocyte phenotypes that exhibited spontaneous depolarizations or spontaneous APs in the PV-LA tissue. Extending the variability of the myocyte phenotype to permit some instances that are spontaneously active (as would be expected of typical myocyte heterogeneity in tissue) is one direction for the framework that could be of obvious value. We did still observe marked changes in tissue electrotonic properties with our excitability simulations. These suggest that the loss-of-function c.932delC (Cx43) mutant is likely to be particularly predisposed to spontaneous focal activity, whereas $I_{\mathrm {Na}}$ loss-of-function accompanying A130V may be sufficient to elicit conduction block in certain conditions. Together, these results reveal that even this initial implementation of the cell-based EMI framework is capable of assessing arrhythmic PV phenotypes involving re-entry, and that more detailed approaches to assessing spontaneous focal activity are likely possible.

### Limitations

Our results suggest that the high-resolution modeling approach permitted by EMI may improve our ability to assess the mechanisms of PV arrhythmogenesis. However, there are clearly aspects that can be improved. First, in this study we have not investigated the potential for microfibrotic regions, which are known to occur at the PV-LA border^64^, to impact conduction and introduce micro-reentry. This would require significantly larger tissue geometries, which while possible are computationally costly, and thus beyond the initial design we have applied here. Second, while we did implement random heterogeneity that was constrained by the mean properties measured in canine LA and PV myocytes^22^, it is likely that real electrophysiologic heterogeneity exceeds these constraints. As a cell-based framework, EMI is uniquely suited to applying more realistic heterogeneity, perhaps by invoking populations of myocyte models constructed from the measured variability in those same canine experiments. Third, we have not considered perturbations to calcium homeostasis in these simulations. Such perturbations are well known to play a role in PV-driven arrhythmia^75^. Fourth, we have assumed that it is sufficient to consider one layer of myocytes. The myocardial thickness of the sleeve of the pulmonary vein varies with the distance from the venoatrial junction. In^62^ the thickness is reported to vary between 0.1 and 2 mm, and from 0.3 to 0.8 mm in^76^. Therefore, the use of only one layer of myocytes is an approximation selected and used in this study for computational tractability. Finally, we also acknowledge that computational cost and the relatively small computational domain used in these simulations have likely limited our ability to address important determinants of PV arrhythmia. In particular, gradients in APD from the PV ostia to the distal PV sleeve have been shown to be important for AF inducibility in this region^12^. While we have implemented local heterogeneities, these systematic gradients exist over larger spatial dimensions than our simulated geometry can allow.

We also highlight two important opportunities to extend this modeling framework:

1. It would be desirable to incorporate diffusive ionic mass conservation in both the extracellular and intracellular spaces to assess longer-term changes to ionic homeostasis in these two domains-currently, the extracellular domain is assumed to be an infinite pool, thus concentrations are constant in space and time. Significant changes in extracellular ionic homeostasis are thought to result from the high frequency AP firing that is characteristic of AF, and explicit implementation of the intracellular and extracellular spaces in EMI offers a unique basis for assessing these effects.
2. It is well known that interplay of parasympathetic and sympathetic inputs are key for inducing PV arrhythmia^66^. However, the degree to which regionalization of these inputs within the PV sleeve promotes arrhythmogenic outcomes is not well understood. Similarly, small regionalized fibrotic borders have been strongly implicated in PV arrhythmia^64^. The high spatial resolution offered by EMI could readily be applied to reveal and study the details of the arrhythmogenic potential both of these very localized effects.

## Conclusions

To our knowledge, this is the first study that has simulated the PV-LA border at a resolution needed to capture mechanisms that operate at a spatial scale of a single myocyte. Our results show that the cell-based EMI framework can discriminate the functional arrhythmogenic influences of known AF mutations in simulations of the PV-LA border. We anticipate that a similar modeling strategy can be applied to investigate the role of defined local microfibrous structures, realistic heterogeneity in myocyte orientation and coupling, and clinically relevant improvement of ablation procedures in this critical PV-LA sleeve region. As such, this modeling framework provides a strong and novel foundation for assessing the unique arrhythmogenic properties of the PV sleeve.

## Supplementary Information

Supplementary Information.

## References

[1] Nattel S, Dobrev D (2017). Controversies about atrial fibrillation mechanisms: Aiming for order in chaos and whether it matters. Circ. Res..

[2] Martignani C, Massaro G, Biffi M, Ziacchi M, Diemberger I (2020). Atrial fibrillation: An arrhythmia that makes healthcare systems tremble. J. Med. Econ..

[3] Feghaly J, Zakka P, London B, MacRae CA, Refaat MM (2018). Genetics of atrial fibrillation. J. Am. Heart Assoc..

[4] Brandes A, Smit MD, Nguyen BO, Rienstra M, Van Gelder IC (2018). Risk factor management in atrial fibrillation. Arrhythm. Electrophysiol. Rev..

[5] Fatkin D, Santiago CF, Huttner IG, Lubitz SA, Ellinor PT (2017). Genetics of atrial fibrillation: State of the art in 2017. Heart Lung Circ..

[6] Weng L-C, Preis SR, Hulme OL, Larson MG, Choi SH, Wang B, Trinquart L, McManus DD, Staerk L, Lin H (2018). Genetic predisposition, clinical risk factor burden, and lifetime risk of atrial fibrillation. Circulation.

[7] Calvo D, Filgueiras-Rama D, Jalife J (2018). Mechanisms and drug development in atrial fibrillation. Pharmacol. Rev..

[8] Adderley NJ, Ryan R, Nirantharakumar K, Marshall T (2019). Prevalence and treatment of atrial fibrillation in UK general practice from 2000 to 2016. Heart.

[9] Koivumäki JT, Korhonen T, Tavi P (2011). Impact of sarcoplasmic reticulum calcium release on calcium dynamics and action potential morphology in human atrial myocytes: a computational study. PLoS Comput. Biol..

[10] Skibsbye L, Jespersen T, Christ T, Maleckar MM, van den Brink J, Tavi P, Koivumäki JT (2016). Refractoriness in human atria: Time and voltage dependence of sodium channel availability. J. Mol. Cell. Cardiol..

[11] Ni H, Fogli Iseppe A, Giles WR, Narayan SM, Zhang H, Edwards AG, Morotti S, Grandi E (2020). Populations of in silico myocytes and tissues reveal synergy of multiatrial-predominant K$$^+$$-current block in atrial fibrillation. Br. J. Pharmacol..

[12] Roney CH, Bayer JD, Cochet H, Meo M, Dubois R, Jaïs P, Vigmond EJ (2018). Variability in pulmonary vein electrophysiology and fibrosis determines arrhythmia susceptibility and dynamics. PLoS Comput. Biol..

[13] Aronis KN, Ali RL, Liang JA, Zhou S, Trayanova NA (2019). Understanding AF mechanisms through computational modelling and simulations. Arrhythm. Electrophysiol. Rev..

[14] Trayanova NA (2014). Mathematical approaches to understanding and imaging atrial fibrillation: Significance for mechanisms and management. Circ. Res..

[15] Clerx, M., Mirams, G. R., Rogers, A. J., Narayan, S. M., Giles, W. R. Immediate and delayed response of simulated human atrial myocytes to clinically-relevant hypokalemia. *Front. Physiol.*, (2021). 10.3389/fphys.2021.651162 PMC8188899 34122128

[16] Aslanidi OV, Colman MA, Varela M, Zhao J, Smaill BH, Hancox JC, Boyett MR, Zhang H (2013). Heterogeneous and anisotropic integrative model of pulmonary veins: Computational study of arrhythmogenic substrate for atrial fibrillation. Interface Focus.

[17] Heijman J, Sutanto H, Crijns HJGM, Nattel S, Trayanova NA (2021). Computational models of atrial fibrillation: Achievements, challenges, and perspectives for improving clinical care. Cardiovasc. Res..

[18] Haissaguerre M, Jaïs P, Shah DC, Takahashi A, Hocini M, Quiniou G, Garrigue S, Le Mouroux A, Le Métayer P, Clémenty J (1998). Spontaneous initiation of atrial fibrillation by ectopic beats originating in the pulmonary veins. N. Engl. J. Med..

[19] Haïssaguerre M, Shah DC, Jaïs P, Hocini M, Yamane T, Deisenhofer I, Chauvin M, Garrigue S, Clémenty J (2000). Electrophysiological breakthroughs from the left atrium to the pulmonary veins. Circulation.

[20] Hocini M, Ho SY, Kawara T, Linnenbank AC, Potse M, Shah D, Jaïs P, Janse MJ, Haïssaguerre M, De Bakker JMT (2002). Electrical conduction in canine pulmonary veins: Electrophysiological and anatomic correlation. Circulation.

[21] Workman AJ, Kane KA, Rankin AC (2008). Cellular bases for human atrial fibrillation. Heart Rhythm.

[22] Ehrlich JR, Cha T-J, Zhang L, Chartier D, Melnyk P, Hohnloser SH, Nattel S (2003). Cellular electrophysiology of canine pulmonary vein cardiomyocytes: Action potential and ionic current properties. J. Physiol..

[23] Varela M, Colman MA, Hancox JC, Aslanidi OV (2016). Atrial heterogeneity generates re-entrant substrate during atrial fibrillation and anti-arrhythmic drug action: Mechanistic insights from canine atrial models. PLoS Comput. Biol..

[24] Anumonwo JM, Pandit SV (2015). Ionic mechanisms of arrhythmogenesis. Trends Cardiovasc. Med..

[25] Jacquemet V, Henriquez CS (2009). Genesis of complex fractionated atrial electrograms in zones of slow conduction: A computer model of microfibrosis. Heart Rhythm.

[26] Franzone, P. C., Pavarino, L. F., Scacchi, S. *Mathematical Cardiac Electrophysiology*, volume 13. Springer, (2014).

[27] Dössel O, Krueger MW, Weber FM, Wilhelms M, Seemann G (2012). Computational modeling of the human atrial anatomy and electrophysiology. Med. Biol. Eng. Comput..

[28] Tveito A, Jæger KH, Kuchta M, Mardal K-A, Rognes ME (2017). A cell-based framework for numerical modeling of electrical conduction in cardiac tissue. Front. Phys..

[29] Jæger KH, Edwards AG, McCulloch A, Tveito A (2019). Properties of cardiac conduction in a cell-based computational model. PLoS Comput. Biol..

[30] Jæger KH, Hustad KG, Cai X, Tveito A (2021). Efficient numerical solution of the EMI model representing the extracellular space (E), cell membrane (M) and intracellular space (I) of a collection of cardiac cells. Front. Phys..

[31] Jæger, K. H., Tveito, A. Derivation of a cell-based mathematical model of excitable cells. In *Modeling Excitable Tissue*, pages 1–13. Springer, Cham, (2020).

[32] Jæger, K. H., Edwards, A. G., Giles, W. R., Tveito, A. From millimeters to micrometers; re-introducing myocytes in models of cardiac electrophysiology. *Front. Physiol.*, **12**, (2021). 10.3389/fphys.2021.763584 PMC8578869 34777021

[33] Spach MS, Heidlage JF, Dolber PC, Barr RC (2007). Mechanism of origin of conduction disturbances in aging human atrial bundles: Experimental and model study. Heart Rhythm.

[34] Niederer S, Mitchell L, Smith N, Plank G (2011). Simulating human cardiac electrophysiology on clinical time-scales. Front. Physiol..

[35] Niederer SA, Kerfoot E, Benson AP, Bernabeu MO, Bernus O, Bradley C, Cherry EM, Clayton R, Fenton FH, Garny A (2011). Verification of cardiac tissue electrophysiology simulators using an n-version benchmark. Philos. Trans. Royal Soc. A Math. Phys. Eng. Sci..

[36] Clayton RH, Panfilov AV (2008). A guide to modelling cardiac electrical activity in anatomically detailed ventricles. Prog. Biophys. Mol. Biol..

[37] Xie F, Zhilin Q, Yang J, Baher A, Weiss JN, Garfinkel A (2004). A simulation study of the effects of cardiac anatomy in ventricular fibrillation. J. Clin. Investig..

[38] Jæger KH, Tveito A (2022). Deriving the bidomain model of cardiac electrophysiology from a cell-based model; properties and comparisons. Front. Physiol..

[39] McPate MJ, Duncan RS, Milnes JT, Witchel HJ, Hancox JC (2005). The N588K-HERG K$$^+$$ channel mutation in the ‘short QT syndrome’: Mechanism of gain-in-function determined at 37 $$^{\circ }$$C. Biochem. Biophys. Res. Commun..

[40] Jæger KH, Wall S, Tveito A (2021). Computational prediction of drug response in short QT syndrome type 1 based on measurements of compound effect in stem cell-derived cardiomyocytes. PLoS Comput. Biol..

[41] Hong KUI, Bjerregaard P, Gussak I, Brugada R (2005). Short QT syndrome and atrial fibrillation caused by mutation in KCNH2. J. Cardiovasc. Electrophysiol..

[42] Olesen MS, Refsgaard L, Holst AG, Larsen AP, Grubb S, Svendsen SHJH, Olesen S-P, Schmitt N, Calloe K (2013). A novel KCND3 gain-of-function mutation associated with early-onset of persistent lone atrial fibrillation. Cardiovasc. Res..

[43] Deo M, Ruan Y, Pandit SV, Shah K, Berenfeld O, Blaufox A, Cerrone M, Noujaim SF, Denegri M, Jalife J, Priori SG (2013). KCNJ2 mutation in short QT syndrome 3 results in atrial fibrillation and ventricular proarrhythmia. Proc. Natl. Acad. Sci..

[44] Olson TM, Alekseev AE, Liu XK, Park S, Zingman LV, Bienengraeber M, Sattiraju S, Ballew JD, Jahangir A, Terzic A (2006). Kv1.5 channelopathy due to KCNA5 loss-of-function mutation causes human atrial fibrillation. Hum. Mol. Genet..

[45] Wang P, Yang Q, Xiaofen W, Yang Y, Shi L, Wang C, Gang W, Xia Y, Yang B, Zhang R (2010). Functional dominant-negative mutation of sodium channel subunit gene SCN3B associated with atrial fibrillation in a chinese GeneID population. Biochem. Biophys. Res. Commun..

[46] Thibodeau IL, Ji X, Li Q, Liu G, Lam K, Veinot JP, Birnie DH, Jones DL, Krahn AD, Lemery R, Nicholson BJ, Gollob MH (2010). Paradigm of genetic mosaicism and lone atrial fibrillation: physiological characterization of a connexin 43-deletion mutant identified from atrial tissue. Circulation.

[47] Jæger KH, Charwat V, Charrez B, Finsberg H, Maleckar MM, Wall S, Healy K, Tveito A (2020). Improved computational identification of drug response using optical measurements of human stem cell derived cardiomyocytes in microphysiological systems. Front. Pharmacol..

[48] Tveito A, Jæger KH, Maleckar MM, Giles WR, Wall S (2020). Computational translation of drug effects from animal experiments to human ventricular myocytes. Sci. Rep..

[49] Jæger KH, Edwards AG, Giles WR, Tveito A (2021). A computational method for identifying an optimal combination of existing drugs to repair the action potentials of SQT1 ventricular myocytes. PLoS Comput. Biol..

[50] Gaita F, Giustetto C, Bianchi F, Wolpert C, Schimpf R, Riccardi R, Grossi S, Richiardi E, Borggrefe M (2003). Short QT syndrome: A familial cause of sudden death. Circulation.

[51] Fink M, Noble D, Virag L, Varro A, Giles WR (2008). Contributions of HERG K$$^{+}$$ current to repolarization of the human ventricular action potential. Prog. Biophys. Mol. Biol..

[52] Grandi E, Pandit SV, Voigt N, Workman AJ, Dobrev D, Jalife J, Bers DM (2011). Human atrial action potential and Ca$$^{2+}$$ model: Sinus rhythm and chronic atrial fibrillation. Circ. Res..

[53] Stinstra, J. G., Roberts, S. F., Pormann, J. B., MacLeod, R. S., Henriquez, C. S. A model of 3D propagation in discrete cardiac tissue. In *Computers in Cardiology*, pages 41–44. IEEE, (2006). PMC1847422 17404606

[54] Stinstra J, MacLeod R, Henriquez C (2010). Incorporating histology into a 3D microscopic computer model of myocardium to study propagation at a cellular level. Ann. Biomed. Eng..

[55] Anderson, R., Andrej, J., Barker, A., Bramwell, J., Camier, J.-S., Cerveny, J., Dobrev,V.,Dudouit, Y., Fisher, A., Kolev, Tz., Pazner, W., Stowell, W., Tomov, V., Akkerman, I., Dahm, J., Medina, D., Zampini, S. MFEM: A modular finite element library. *Comput. Math. Appl.*, (2020).

[56] MFEM: Modular finite element methods [Software]. mfem.org.

[57] Jæger, K. H., Hustad, K. G., Cai, X., Tveito, A.. Operator splitting and finite difference schemes for solving the EMI model. In *Modeling Excitable Tissue*, pages 44–55. Springer, Cham, (2020).

[58] Yamane T, Shah DC, Jaïs P, Hocini M, Peng JT, Deisenhofer I, Clémenty J, Haïssaguerre M (2002). Dilatation as a marker of pulmonary veins initiating atrial fibrillation. J. Interv. Card. Electrophysiol..

[59] Nygren A, Fiset C, Firek L, Clark JW, Lindblad DS, Clark RB, Giles WR (1998). Mathematical model of an adult human atrial cell: The role of K$$^+$$ currents in repolarization. Circ. Res..

[60] Xie F, Zhilin Q, Garfinkel A, Weiss JN (2002). Electrical refractory period restitution and spiral wave reentry in simulated cardiac tissue. Am. J. Physiol.-Heart Circ. Physiol..

[61] Ludatscher RM (1968). Fine structure of the muscular wall of rat pulmonary veins. J. Anat..

[62] Ho SY, Cabrera JA, Tran VH, Farre J, Anderson RH, Sanchez-Quintana D (2001). Architecture of the pulmonary veins: Relevance to radiofrequency ablation. Heart.

[63] Ho SY, Sanchez-Quintana D, Cabrera JA, Anderson RH (1999). Anatomy of the left atrium: Implications for radiofrequency ablation of atrial fibrillation. J. Cardiovasc. Electrophysiol..

[64] Hamabe A, Okuyama Y, Miyauchi Y, Zhou S, Pak H-N, Karagueuzian HS, Fishbein MC, Chen P-S (2003). Correlation between anatomy and electrical activation in canine pulmonary veins. Circulation.

[65] Colman MA, Aslanidi OV, Kharche S, Boyett MR, Garratt C, Hancox JC, Zhang H (2013). Pro-arrhythmogenic effects of atrial fibrillation-induced electrical remodelling: Insights from the three-dimensional virtual human atria. J. Physiol..

[66] Patterson E, Po SS, Scherlag BJ, Lazzara R (2005). Triggered firing in pulmonary veins initiated by in vitro autonomic nerve stimulation. Heart Rhythm.

[67] Patterson E, Lazzara R, Szabo B, Liu H, Tang D, Li Y-H, Scherlag BJ, Po SS (2006). Sodium-calcium exchange initiated by the ca2+ transient: An arrhythmia trigger within pulmonary veins. J. Am. Coll. Cardiol..

[68] Perez-Lugones A, McMahon JT, Ratliff NB, Saliba WI, Schweikert RA, Marrouche NF, Saad EB, Navia JL, McCarthy PM, Tchou P (2003). Evidence of specialized conduction cells in human pulmonary veins of patients with atrial fibrillation. J. Cardiovasc. Electrophysiol..

[69] Cherry EM, Ehrlich JR, Nattel S, Fenton FH (2007). Pulmonary vein reentry–properties and size matter: Insights from a computational analysis. Heart Rhythm.

[70] Gong Y, Xie F, Stein KM, Garfinkel A, Culianu CA, Lerman BB, Christini DJ (2007). Mechanism underlying initiation of paroxysmal atrial flutter/atrial fibrillation by ectopic foci: A simulation study. Circulation.

[71] Hwang M, Lim B, Song J-S, Hee Tae Yu, Ryu A-J, Lee Y-S, Joung B, Shim EB, Pak H-N (2017). Ganglionated plexi stimulation induces pulmonary vein triggers and promotes atrial arrhythmogenecity: In silico modeling study. PLoS ONE.

[72] Colman MA, Varela M, Hancox JC, Zhang H, Aslanidi OV (2014). Evolution and pharmacological modulation of the arrhythmogenic wave dynamics in canine pulmonary vein model. Europace.

[73] Aslanidi OV, Colman MA, Stott J, Dobrzynski H, Boyett MR, Holden AV, Zhang H (2011). 3D virtual human atria: A computational platform for studying clinical atrial fibrillation. Prog. Biophys. Mol. Biol..

[74] Shade JK, Ali RL, Basile D, Popescu D, Akhtar T, Marine JE, Spragg DD, Calkins H, Trayanova NA (2020). Preprocedure application of machine learning and mechanistic simulations predicts likelihood of paroxysmal atrial fibrillation recurrence following pulmonary vein isolation. Circ. Arrhythm. Electrophysiol..

[75] Stavrakis S, Po S (2017). Ganglionated plexi ablation: Physiology and clinical applications. Arrhythm. Electrophysiol. Rev..

[76] Hassink RJ, Aretz HT, Ruskin J, Keane D (2003). Morphology of atrial myocardium in human pulmonary veins: A postmortem analysis in patients with and without atrial fibrillation. J. Am. Coll. Cardiol..

