# Supplementary material for: Arrhythmogenic influence of mutations in a myocyte-based computational model of the pulmonary vein sleeve
Source: Sci Rep. 2022 Apr 29;12:7040. doi: 10.1038/s41598-022-11110-1 (PMC9054808; doi:10.1038/s41598-022-11110-1)
Supplement: Supplementary file 1 — Supplementary Information. [file 41598_2022_11110_MOESM1_ESM.pdf]

Supplementary Information for *Arrhythmogenic Influence of Mutations in a Myocyte-Based Computational Model of the Pulmonary Vein Sleeve*

Karoline Horgmo Jæger[1], Andrew G. Edwards[1], Wayne R. Giles[1,2], and Aslak Tveito[1]

[1]Simula Research Laboratory, Norway
[2]Department of Physiology and Pharmacology, Cumming School of Medicine, University of Calgary, Calgary, Canada

Contents

S1 Base model formulation **2**
S1.1 Membrane potential 2
S1.2 Transmembrane currents 2
S1.3 Intracellular [Ca^{2+}] dynamics 7
S1.4 Ca^{2+} fluxes 8
S1.5 Intracellular Na^{+} dynamics 9
S1.6 Nernst equilibrium potentials 9
S1.7 Baseline parameter values 9

S2 Supplementary figures **15**
Figure S1: Comparison of the LA base model AP to other human atrial AP models 15
Figure S2: Comparison of the LA base model currents to other human atrial AP models 16
Figure S3: Currents at rest for the LA and PV base models 17
Figure S4: Voltage dependence of WT and E299V I_{K1} 17

S3 Wavelength and re-entry **18**

S1 Base model formulation

In this supplementary section, we describe the formulation of the human left atrial and pulmonary vein myocyte versions of our base model. Here, the membrane potential (v) is given in units of mV, and the Ca^{2+} and Na^{+} concentrations are given in units of mM. All currents are given in units of A/F, and the ionic fluxes are expressed as mmol/ms per total cell volume (i.e., in units of mM/ms). Time is given in ms. The parameters of the model are all given in Tables S1–S7. In particular, the adjustment factors used to scale the model from the left atrial version to the pulmonary vein version of the model are found in Table S4.

Note that the model formulation is very similar to the base model from [1, 2]. The main difference is that the I_{Kur} current is included and that the conductances of the currents and fluxes are adjusted to represent atrial cardiomyocytes.

S1.1 Membrane potential

In the base model formulation, the membrane potential is governed by

$$\begin{aligned}\frac{dv}{dt} = -(&I_{\mathrm{Na}} + I_{\mathrm{NaL}} + I_{\mathrm{CaL}} + I_{\mathrm{to}} + I_{\mathrm{Kr}} + I_{\mathrm{Ks}} + I_{\mathrm{K1}} + I_{\mathrm{NaCa}} + I_{\mathrm{NaK}} \\ &+ I_{\mathrm{pCa}} + I_{\mathrm{bCa}} + I_{\mathrm{bCl}} + I_{\mathrm{bNa}} + I_{\mathrm{f}} + I_{\mathrm{Kur}} + I_{\mathrm{stim}}),\end{aligned} \tag{1}$$

where I_{Na}, I_{NaL}, I_{CaL}, I_{to}, I_{Kr}, I_{Ks}, I_{K1}, I_{NaCa}, I_{NaK}, I_{pCa}, I_{bCl}, I_{bCa}, I_{bNa}, I_{f}, and I_{Kur} are transmembrane currents that will be specified below and I_{stim} is an applied stimulus current. Unless otherwise specified, we let I_{stim} be given as a constant current of size -40 A/F applied until the membrane potential reaches a value of -40 mV.

S1.2 Transmembrane currents

In general, the currents through the voltage-gated ion channels within the myocyte membrane are given on the form

$$I = go(v - E),$$

where g is the channel conductance, v is the membrane potential and E is the equilibrium potential of the channel. Moreover, o is the open probability of the channels, which is given on the form $o = \prod_i z_i$, where z_i are gating variables. These gating variables are either given as an explicit function of

the membrane potential or governed by equations of the form

$$z_i' = \frac{1}{\tau_{z_i}}(z_{i,\infty} - z_i). \tag{2}$$

The parameters τ_{z_i} and $z_{i,\infty}$ are specified for each of the gating variables of the model in Table S9.

Fast sodium current (I_{Na}) The formulation of the fast sodium current is based on the model formulation given in [3] and is given by

$$I_{\mathrm{Na}} = g_{\mathrm{Na}} o_{\mathrm{Na}}(v - E_{\mathrm{Na}}), \tag{3}$$

where the open probability is given by

$$o_{\mathrm{Na}} = m^3 j, \tag{4}$$

and m and j are gating variables governed by equations of the form (2).

Late sodium current (I_{NaL}) The formulation of the late sodium current, I_{NaL}, is based on [4] and is given by

$$I_{\mathrm{NaL}} = g_{\mathrm{NaL}} o_{\mathrm{NaL}}(v - E_{\mathrm{Na}}), \tag{5}$$

where the open probability is given by

$$o_{\mathrm{NaL}} = m_L h_L, \tag{6}$$

and m_L and h_L are gating variables governed by equations of the form (2).

Transient outward potassium current (I_{to}) The formulation of the transient outward potassium current, I_{to}, is based on [5] and is given by

$$I_{\mathrm{to}} = g_{\mathrm{to}} o_{\mathrm{to}}(v - E_{\mathrm{K}}), \tag{7}$$

where the open probability is given by

$$o_{\mathrm{to}} = q_{\mathrm{to}} r_{\mathrm{to}}, \tag{8}$$

and q_{to} and r_{to} are gating variables governed by equations of the form (2). For the A545P mutation, g_{to} is increased by 75% and $\tau_{r_{\mathrm{to}}}$ is increased by 15%.

Rapidly activating potassium current (I_{Kr}) The rapidly activating potassium current, I_{Kr}, is formulated as a Markov model, based on [6]. The formulation has been fitted to data of WT and N588K I_{Kr} currents from [7]. The current is given by

$$I_{\mathrm{Kr}} = g_{\mathrm{Kr}} \sqrt{\frac{[\mathrm{K}^+]_e}{[\mathrm{K}^+]_e^b}} \frac{T - 275 \text{ K}}{35 \text{ K}} o_{\mathrm{Kr}} (v - E_K), \tag{9}$$

where o_{Kr} is modelled by a Markov model of the form

$$c_{\mathrm{Kr},1} \underset{\beta_{\mathrm{Kr},1}}{\overset{\alpha_{\mathrm{Kr},1}}{\rightleftharpoons}} c_{\mathrm{Kr},2} \underset{\beta_{\mathrm{Kr},2}}{\overset{\alpha_{\mathrm{Kr},2}}{\rightleftharpoons}} c_{\mathrm{Kr},3} \underset{\beta_{\mathrm{Kr},3}}{\overset{\alpha_{\mathrm{Kr},3}}{\rightleftharpoons}} o_{\mathrm{Kr}} \underset{\beta_{\mathrm{Kr},4}}{\overset{\alpha_{\mathrm{Kr},4}}{\rightleftharpoons}} i_{\mathrm{Kr}}. \tag{10}$$

Here, the dynamics of the closed states $c_{\mathrm{Kr},1}$, $c_{\mathrm{Kr},2}$, and $c_{\mathrm{Kr},3}$, the open state o_{Kr}, and the inactivated state i_{Kr} are given by

$$\frac{c_{\mathrm{Kr},1}}{dt} = \beta_{\mathrm{Kr},1} c_{\mathrm{Kr},2} - \alpha_{\mathrm{Kr},1} c_{\mathrm{Kr},1}, \tag{11}$$

$$\frac{c_{\mathrm{Kr},2}}{dt} = \alpha_{\mathrm{Kr},1} c_{\mathrm{Kr},1} + \beta_{\mathrm{Kr},2} c_{\mathrm{Kr},3} - (\alpha_{\mathrm{Kr},2} + \beta_{\mathrm{Kr},1}) c_{\mathrm{Kr},2}, \tag{12}$$

$$\frac{c_{\mathrm{Kr},3}}{dt} = \alpha_{\mathrm{Kr},2} c_{\mathrm{Kr},2} + \beta_{\mathrm{Kr},3} o_{\mathrm{Kr}} - (\alpha_{\mathrm{Kr},3} + \beta_{\mathrm{Kr},2}) c_{\mathrm{Kr},3}, \tag{13}$$

$$\frac{o_{\mathrm{Kr}}}{dt} = \alpha_{\mathrm{Kr},3} c_{\mathrm{Kr},3} + \beta_{\mathrm{Kr},4} i_{\mathrm{Kr}} - (\alpha_{\mathrm{Kr},4} + \beta_{\mathrm{Kr},3}) o_{\mathrm{Kr}}, \tag{14}$$

$$\frac{i_{\mathrm{Kr}}}{dt} = \alpha_{\mathrm{Kr},4} o_{\mathrm{Kr}} - \beta_{\mathrm{Kr},4} i_{\mathrm{Kr}}, \tag{15}$$

where the transition rates are given by provided in Table S8. The N588K mutation is represented in the model by multiplying $\alpha_{\mathrm{Kr},4}$ and $\beta_{\mathrm{Kr},4}$ by 0.25 and 5, respectively.

Slowly activating potassium current (I_{Ks}) The formulation of the slowly activating potassium current, I_{Ks}, is based on [3] and is given by

$$I_{\mathrm{Ks}} = g_{\mathrm{Ks}} o_{\mathrm{Ks}} (v - E_{\mathrm{Ks}}), \tag{16}$$

where

$$o_{\mathrm{Ks}} = x_{\mathrm{Ks}}^2, \tag{17}$$

and the dynamics of x_{Ks} is governed by an equation of the form (2).

Inward rectifier potassium current (I_{K1}) The formulation of the inward rectifier potassium current, I_{K1}, is based on [8, 9], and is given by

$$I_{K1} = g_{K1}\sqrt{\frac{[K^+]_e}{[K^+]_e^b}}\left(\frac{0.059764(v - E_K + 0.582854)}{0.754829 + e^{0.0767156(v-E_K+0.582854)}} - 0.0114237\right) \qquad \text{(WT)},$$

$$I_{K1} = g_{K1}\sqrt{\frac{[K^+]_e}{[K^+]_e^b}}\big(0.0166814(v - E_K + 0.0118494)$$
$$- 6.6243 \cdot 10^{-5}(v - E_K + 0.0118494)^2 + 0.034651\big) \qquad \text{(E299V)},$$

for wild type and the E299V mutation, respectively.

Ultrarapid delayed rectifier potassium current (I_{Kur}) The formulation of the ultrarapid delayed rectifier potassium current, I_{Kur}, is based on [10, 11] and is given by

$$I_{Kur} = g_{Kur} f_{Kur} o_{Kur}(v - E_K), \tag{18}$$

where f_{Kur} is given by

$$f_{Kur} = 0.005 + \frac{0.05}{1 + e^{-\frac{v-15}{13}}} \tag{19}$$

and

$$o_{Kur} = x_{Kur1}^3 \cdot x_{Kur2}. \tag{20}$$

The dynamics of x_{Kur1} and x_{Kur2} are governed by equations of the form (2). For the E375X mutation g_{Kur} is reduced by 90%.

Hyperpolarization activated funny current (I_f) The formulation for the hyperpolarization activated funny current, I_f, is based on [5] and is given by

$$I_f = g_f o_f (v - E_f), \tag{21}$$

where

$$o_f = x_f, \tag{22}$$

and the dynamics of x_f is governed by an equation of the form (2).

L-type Ca^{2+} current (I_{CaL}) The formualtion for the L-type Ca^{2+} current, I_{CaL}, is based on the formulation in [3] and is given by

$$I_{\text{CaL}} = g_{\text{CaL}} \left(Q_{10}^{\text{CaL}}\right)^{Q_p} o_{\text{CaL}} \frac{(2F)^2 v}{RT} \frac{0.341 c_d e^{\frac{2Fv}{RT}} - 0.341 c_e}{e^{\frac{2Fv}{RT}} - 1}, \tag{23}$$

where

$$o_{\text{CaL}} = df(1 - f_{\text{Ca}}), \tag{24}$$

and the dynamics of d, f and f_{Ca} are governed by equations of the form (2). Note that compared to earlier versions of the base model, the dynamics of the d gate have now been adjusted in order for the current to behave similarly to other human published atrial membrane models [10, 12, 13].

Time-independent background currents (I_{bCa}, I_{bBa}, I_{bCl}) The formulation of the background currents, I_{bCa}, I_{bNa} and I_{bCl}, are based on [3] and are given by

$$I_{\text{bCa}} = g_{\text{bCa}}(v - E_{\text{Ca}}), \tag{25}$$

$$I_{\text{bNa}} = g_{\text{bNa}}(v - E_{\text{Na}}), \tag{26}$$

$$I_{\text{bCl}} = g_{\text{bCl}}(v - E_{\text{Cl}}). \tag{27}$$

Sodium-calcium exchanger current (I_{NaCa}) The formulation of the Na^+-Ca^{2+} exchanger current, I_{NaCa}, is based on [3] and is given by

$$I_{\text{NaCa}} = \bar{I}_{\text{NaCa}} \left(Q_{10}^{\text{NaCa}}\right)^{Q_p} \frac{e^{\frac{\nu F v}{RT}} [\text{Na}^+]_i^3 c_e - e^{\frac{(\nu-1)Fv}{RT}} [\text{Na}^+]_e^3 c_{sl}}{s_{\text{NaCa}} \left(1 + \left(\frac{K_{\text{act}}}{c_{sl}}\right)^2\right) \left(1 + k_{\text{sat}} e^{\frac{(\nu-1)Fv}{RT}}\right)}, \tag{28}$$

where

$$s_{\text{NaCa}} = K_{\text{Ca},i} [\text{Na}^+]_e^3 \left(1 + \left(\frac{[\text{Na}^+]_i}{K_{\text{Na},i}}\right)^3\right) + K_{\text{Na},e}^3 c_{sl} \left(1 + \frac{c_{sl}}{K_{\text{Ca},i}}\right) + K_{\text{Ca},e} [\text{Na}^+]_i^3 + [\text{Na}^+]_i^3 c_e + [\text{Na}^+]_e^3 c_{sl}.$$

Sarcolemmal Ca^{2+} pump current (I_{pCa}) The formulation of the current through the sarcolemmal Ca^{2+} pump, I_{pCa}, is based on [3] and is given by

$$I_{\text{pCa}} = \bar{I}_{\text{pCa}} \left(Q_{10}^{\text{pCa}}\right)^{Q_p} \frac{c_{sl}^2}{K_{\text{pCa}}^2 + c_{sl}^2}. \tag{29}$$

Sodium-potassium pump current (I_{NaK}) The current through the Na^{+}-K^{+} pump, I_{NaK}, is based on [3] and is given by

$$I_{\mathrm{NaK}} = \bar{I}_{\mathrm{NaK}} \left(Q_{10}^{\mathrm{NaK}}\right)^{Q_p} \frac{f_{\mathrm{NaK}}}{1 + \left(\frac{K_{\mathrm{Na,i}}^{\mathrm{NaK}}}{[\mathrm{Na}^+]_{\mathrm{i}}}\right)^4} \frac{[\mathrm{K}^+]_{\mathrm{e}}}{[\mathrm{K}^+]_{\mathrm{e}} + K_{\mathrm{K,e}}}, \tag{30}$$

where

$$f_{\mathrm{NaK}} = \frac{1}{1 + 0.12e^{-0.1\frac{Fv}{RT}}} + \frac{0.037}{7}\left(e^{\frac{[\mathrm{Na}^+]_{\mathrm{e}}}{67}} - 1\right) e^{-\frac{Fv}{RT}}. \tag{31}$$

S1.3 Intracellular [Ca^{2+}] dynamics

The Ca^{2+} dynamics are governed by

$$\frac{dc_d}{dt} = \frac{1}{V_d}(J_{\mathrm{CaL}} - J_d^b - J_d^c), \qquad \frac{db_d}{dt} = \frac{1}{V_d}J_d^b, \tag{32}$$

$$\frac{dc_{sl}}{dt} = \frac{1}{V_{sl}}(J_e^{sl} - J_{sl}^c - J_{sl}^b + J_s^{sl}), \qquad \frac{db_{sl}}{dt} = \frac{1}{V_{sl}}J_{sl}^b, \tag{33}$$

$$\frac{dc_c}{dt} = \frac{1}{V_c}(J_{sl}^c + J_d^c - J_c^n - J_c^b), \qquad \frac{db_c}{dt} = \frac{1}{V_c}J_c^b, \tag{34}$$

$$\frac{dc_s}{dt} = \frac{1}{V_s}(J_n^s - J_s^{sl} - J_s^b), \qquad \frac{db_s}{dt} = \frac{1}{V_s}J_s^b, \tag{35}$$

$$\frac{dc_n}{dt} = \frac{1}{V_n}(J_c^n - J_n^s). \tag{36}$$

Here, c_d is the concentration of free Ca^{2+} in the dyad, b_d is the concentration of Ca^{2+} bound to a buffer in the dyad, c_{sl} is the concentration of free Ca^{2+} in the sub-sarcolemmal (SL) compartment, b_{sl} is the concentration of Ca^{2+} bound to a buffer in the SL compartment, c_c is the concentration of free Ca^{2+} in the bulk cytosol, b_c is the concentration of Ca^{2+} bound to a buffer in the bulk cytosol, c_s is the concentration of free Ca^{2+} in the junctional sarcoplasmic reticulum (jSR), b_s is the concentration of Ca^{2+} bound to a buffer in the jSR, and c_n is the concentration of free Ca^{2+} in the network sarcoplasmic reticulum (nSR). The expressions for the fluxes are specified below.

S1.4 Ca^{2+} fluxes

Flux through the SERCA pumps The flux from the bulk cytosol to the nSR through the SERCA pumps is based on [3] and given by

$$J_c^n = \bar{J}_{\mathrm{SERCA}} \left(Q_{10}^{\mathrm{SERCA}}\right)^{Q_p} \frac{\left(\frac{c_c}{K_c}\right)^2 - \left(\frac{c_n}{K_n}\right)^2}{1 + \left(\frac{c_c}{K_c}\right)^2 + \left(\frac{c_n}{K_n}\right)^2}. \tag{37}$$

Flux through the RyRs The flux from the jSR to the SL compartment is given by

$$J_s^{sl} = J_{\mathrm{RyR}} + J_{\mathrm{leak}}, \tag{38}$$

where J_{RyR} is the flux through the active RyR channels and J_{leak} is the flux through passive RyR channels that are always open, given by

$$J_{\mathrm{RyR}} = p \cdot r \cdot \alpha_{\mathrm{RyR}}(c_s - c_{sl}), \tag{39}$$

$$J_{\mathrm{leak}} = \gamma_{\mathrm{RyR}} \cdot \alpha_{\mathrm{RyR}}(c_s - c_{sl}), \tag{40}$$

respectively. Here, p represents the open probability of the active RyR channels and is given by

$$p = \frac{c_d^3}{c_d^3 + \kappa_{\mathrm{RyR}}^3}. \tag{41}$$

Furthermore, r is the fraction of RyR channels that are not inactivated and is governed by the equation

$$\frac{dr}{dt} = -\frac{J_{\mathrm{RyR}}}{\beta_{\mathrm{RyR}}} + \frac{\eta_{\mathrm{RyR}}}{p}(1 - r). \tag{42}$$

Passive diffusion fluxes between compartments The passive diffusion fluxes between intracellular compartments are given by

$$J_d^c = \alpha_d^c(c_d - c_c), \tag{43}$$

$$J_{sl}^c = \alpha_{sl}^c(c_{sl} - c_c), \tag{44}$$

$$J_n^s = \alpha_n^s(c_n - c_s). \tag{45}$$

Buffer fluxes The fluxes of free Ca^{2+} binding to a Ca^{2+} buffer are given by

$$J_d^b = V_d(k_{\mathrm{on}}^d c_d(B_{\mathrm{tot}}^d - b_d) - k_{\mathrm{off}}^d b_d), \tag{46}$$

$$J_{sl}^b = V_{sl}(k_{\mathrm{on}}^{sl} c_{sl}(B_{\mathrm{tot}}^{sl} - b_{sl}) - k_{\mathrm{off}}^{sl} b_{sl}), \tag{47}$$

$$J_c^b = V_c(k_{\mathrm{on}}^c c_c(B_{\mathrm{tot}}^c - b_c) - k_{\mathrm{off}}^c b_c), \tag{48}$$

$$J_s^b = V_s(k_{\mathrm{on}}^s c_s(B_{\mathrm{tot}}^s - b_s) - k_{\mathrm{off}}^s b_s). \tag{49}$$

Membrane fluxes The membrane Ca^{2+} fluxes, J_{CaL}, J_{bCa}, J_{pCa}, and J_{NaCa}, are given by

$$J_{\mathrm{CaL}} = -\frac{\chi C_m}{2F} I_{\mathrm{CaL}}, \qquad J_{\mathrm{pCa}} = -\frac{\chi C_m}{2F} I_{\mathrm{pCa}}, \tag{50}$$

$$J_{\mathrm{bCa}} = -\frac{\chi C_m}{2F} I_{\mathrm{bCa}}, \qquad J_{\mathrm{NaCa}} = \frac{\chi C_m}{F} I_{\mathrm{NaCa}}, \tag{51}$$

where I_{CaL}, I_{bCa}, I_{pCa}, and I_{NaCa} are defined by the expressions given above. Furthermore,

$$J_e^{sl} = J_{\mathrm{NaCa}} + J_{\mathrm{pCa}} + J_{\mathrm{bCa}}. \tag{52}$$

S1.5 Intracellular Na^+ dynamics

The intracellular Na^+ concentration is governed by

$$\frac{d[\mathrm{Na}_i]}{dt} = -\frac{\chi C_m}{F} \left(I_{\mathrm{Na}} + I_{\mathrm{NaL}} + I_{\mathrm{bNa}} + 3I_{\mathrm{NaK}} + 3I_{\mathrm{NaCa}} + 0.3293 I_{\mathrm{f}} \right), \tag{53}$$

where the currents I_{Na}, I_{NaL}, I_{bNa}, I_{NaK}, I_{NaCa}, and I_{f} are specified above.

S1.6 Nernst equilibrium potentials

The Nernst equilibrium potentials for the ion channels are defined as

$$E_{\mathrm{Na}} = \frac{RT}{F} \log \left(\frac{[\mathrm{Na}^+]_{\mathrm{e}}}{[\mathrm{Na}^+]_{\mathrm{i}}} \right), \tag{54}$$

$$E_{\mathrm{Ca}} = \frac{RT}{2F} \log \left(\frac{[\mathrm{Ca}^{2+}]_{\mathrm{e}}}{c_{sl}} \right), \tag{55}$$

$$E_{\mathrm{K}} = \frac{RT}{F} \log \left(\frac{[\mathrm{K}^+]_{\mathrm{e}}}{[\mathrm{K}^+]_{\mathrm{i}}} \right), \tag{56}$$

$$E_{\mathrm{Ks}} = \frac{RT}{F} \log \left(\frac{[\mathrm{K}^+]_{\mathrm{e}} + 0.018[\mathrm{Na}^+]_{\mathrm{e}}}{[\mathrm{K}^+]_{\mathrm{i}} + 0.018[\mathrm{Na}^+]_{\mathrm{i}}} \right), \tag{57}$$

$$E_{\mathrm{Cl}} = \frac{RT}{F} \log \left(\frac{[\mathrm{Cl}^+]_{\mathrm{e}}}{[\mathrm{Cl}^+]_{\mathrm{i}}} \right), \tag{58}$$

$$E_{\mathrm{f}} = -17 \text{ mV}, \tag{59}$$

for the parameter values given in Table S2.

S1.7 Baseline parameter values

Parameter	Description	Value
V_d	Volume fraction of the dyadic subspace	0.001
V_{sl}	Volume fraction of the SL compartment	0.028
V_c	Volume fraction of the bulk cytosol	0.917
V_s	Volume fraction of the jSR	0.004
V_n	Volume fraction of the nSR	0.05
χ	Cell surface to volume ratio	0.6 μm^{-1}

Table S1: Default geometry parameters of the base model.

Parameter	Description	Value
C_m	Specific membrane capacitance	0.01 pF/μm^2
F	Faraday's constant	96.485 C/mmol
R	Universal gas constant	8.314 J/(mol·K)
T	Temperature	310 K
$[Ca^{2+}]_e$	Extracellular Ca^{2+} concentration	1.8 mM
$[Na^+]_e$	Extracellular sodium concentration	140 mM
$[K^+]_e$	Extracellular potassium concentration	5.4 mM
$[K^+]_e^b$	Baseline extracellular potassium concentration	5.4 mM
$[K^+]_i$	Intracellular potassium concentration	120 mM
$[Cl^-]_e$	Extracellular chloride concentration	150 mM
$[Cl^-]_i$	Intracellular chloride concentration	15 mM

Table S2: Physical constants and ionic concentrations of the base model.

Parameter	Value	Parameter	Value
g_{Na}	7.056 mS/μF	$\bar{I}_{\text{NaCa}}$	2.22 μA/μF
g_{NaL}	0.021 mS/μF	$\bar{I}_{\text{pCa}}$	0.068 μA/μF
g_{to}	0.55 mS/μF	$\bar{J}_{\text{SERCA}}$	0.00024 mM/ms
g_{Kr}	1.2 mS/μF	α_{RyR}	0.0075 ms^{-1}
g_{Ks}	0.21 mS/μF	β_{RyR}	0.038 mM
g_{K1}	2.62 mS/μF	α_d^c	0.0034 ms^{-1}
g_{f}	0.0001 mS/μF	α_{sl}^c	0.15 ms^{-1}
g_{bCl}	0.0114 mS/μF	α_n^s	0.012 ms^{-1}
g_{CaL}	0.049 nL/(μF ms)	B_{tot}^c	0.07 mM
g_{bCa}	0.000832 mS/μF	B_{tot}^d	1.2 mM
g_{bNa}	0.00185 mS/μF	B_{tot}^{sl}	0.9 mM
g_{Kur}	2.475 mS/μF	B_{tot}^s	27 mM
$\bar{I}_{\text{NaK}}$	2.76 μA/μF		

Table S3: Conductances and similar cell-specific parameter values in the base model formulation. Note that the parameter values of this table define the human left atrial version of the base model. For the human pulmonary vein version, the adjustment factors of Table S4 are applied.

Parameter	PV scaling factor
g_{K1}	0.58
g_{Kr}	1.5
g_{Ks}	1.6
g_{to}	0.75
g_{CaL}	0.7

Table S4: Adjustment factors for the pulmonary vein (PV) version of the base model, based on [14].

Parameter	Flux	Value
K_c	J_c^n	0.00025 mM
K_n	J_c^n	1.7 mM
γ_{RyR}	J_s^{sl}	0.001
κ_{RyR}	J_{RyR}	0.015 mM
η_{RyR}	J_s^{sl}	0.00001 ms^{-1}

Table S5: Parameters for the intracellular Ca^{2+} fluxes of the base model.

Parameter	Current	Value
k_{sat}	I_{NaCa}	0.3
ν	I_{NaCa}	0.3
K_{act}	I_{NaCa}	0.00015 mM
$K_{\mathrm{Ca},i}$	I_{NaCa}	0.0036 mM
$K_{\mathrm{Ca},e}$	I_{NaCa}	1.3 mM
$K_{\mathrm{Na},i}$	I_{NaCa}	12.3 mM
$K_{\mathrm{Na},e}$	I_{NaCa}	87.5 mM
$K_{\mathrm{Na},i}^{\mathrm{NaK}}$	I_{NaK}	$(11\ \mathrm{mM})\cdot(Q_{10}^{\mathrm{KNaK}})^{Q^p}$
$K_{\mathrm{K},e}$	I_{NaK}	1.5 mM
K_{pCa}	I_{pCa}	0.0005 mM

Table S6: Additional parameters for the membrane currents of the base model.

Parameter	Compartment	Value
k_{on}^c	Bulk cytosol	40 $\mathrm{ms}^{-1}\mathrm{mM}^{-1}$
k_{off}^c	Bulk cytosol	0.03 ms^{-1}
k_{on}^d	Dyad	100 $\mathrm{ms}^{-1}\mathrm{mM}^{-1}$
k_{off}^d	Dyad	1 ms^{-1}
k_{on}^{sl}	Subsarcolemmal space	100 $\mathrm{ms}^{-1}\mathrm{mM}^{-1}$
k_{off}^{sl}	Subsarcolemmal space	0.15 ms^{-1}
k_{on}^s	Junctional SR	100 $\mathrm{ms}^{-1}\mathrm{mM}^{-1}$
k_{off}^s	Junctional SR	65 ms^{-1}

Table S7: Transition rates for the Ca^{2+} buffers of the base model.

$\alpha_{\text{Kr},1}$	$0.4 \cdot T_* \cdot e^{24.335+T_*^{-1}(0.0112v-25.914)}$
$\beta_{\text{Kr},1}$	$T_* \cdot e^{13.688+T_*^{-1}(-0.0603v-15.707)}$
$\alpha_{\text{Kr},2}$	$0.4 \cdot T_* \cdot e^{22.746+T_*^{-1}(-25.914)}$
$\beta_{\text{Kr},2}$	$T_* \cdot e^{13.193+T_*^{-1}(-15.707)}$
$\alpha_{\text{Kr},3}$	$0.4 \cdot T_* \cdot e^{22.098+T_*^{-1}(0.0365v-25.914)}$
$\beta_{\text{Kr},3}$	$T_* \cdot e^{7.313+T_*^{-1}(-0.0399v-15.707)}$
$\alpha_{\text{Kr},4}$ (WT)	$T_* \cdot \left(\frac{[\text{K}^+]_e^b}{[\text{K}^+]_e}\right)^{0.4} e^{30.016+T_*^{-1}(0.0223v-30.888)}$
$\alpha_{\text{Kr},4}$ (N588K)	$0.25 \cdot T_* \cdot \left(\frac{[\text{K}^+]_e^b}{[\text{K}^+]_e}\right)^{0.4} e^{30.016+T_*^{-1}(0.0223v-30.888)}$
$\beta_{\text{Kr},4}$ (WT)	$T_* \cdot e^{30.061+T_*^{-1}(-0.0312v-33.243)}$
$\beta_{\text{Kr},4}$ (N588K)	$5 \cdot T_* \cdot e^{30.061+T_*^{-1}(-0.0312v-33.243)}$

Table S8: Transition rates for the I_{Kr} Markov model. Here, $T_* = \frac{T}{310 \text{ K}}$, where T is the temperature.

Current	Gate	z_∞	α_z	β_z	τ_z
I_{Na}	m	$\frac{1}{(1+e^{(-57-v)/9})^2}$	$0.13e^{-((v+46)/16)^2}$	$0.06e^{-((v-5)/51)^2}$	$\frac{\alpha_m+\beta_m}{(Q_{10}^{\text{Na}})^{Q_p}}$
	j	$\frac{1}{(1+e^{(v+72)/7})^2}$	0, if $v \geq -40$; $\frac{\left(-2.5\cdot 10^4 e^{0.2v} - 7\cdot 10^{-6}e^{-0.04v}\right)(v+38)}{1+e^{0.3(v+79)}}$, otherwise	$\frac{0.6e^{0.06v}}{1+e^{-0.1(v+32)}}$, if $v \geq -40$; $\frac{0.02e^{-0.01v}}{1+e^{-0.14(v+40)}}$, otherwise	$\frac{1}{(\alpha_j+\beta_j)(Q_{10}^{\text{Na}})^{Q_p}}$
I_{NaL}	m_L	$\frac{1}{1+e^{(-43-v)/5}}$	$\frac{1}{6.8e^{(v+12)/35}}$	$8.6e^{-(v+77)/6}$	$\frac{\alpha_m+\beta_m}{(Q_{10}^{\text{NaL}})^{Q_p}}$
	h_L	$\frac{1}{1+e^{(v+88)/7.5}}$			$\frac{200 \text{ ms}}{(Q_{10}^{\text{NaL}})^{Q_p}}$
I_{CaL}	d	$\frac{1}{1+e^{-(v+20)/6}}$	$\frac{1-e^{-\frac{v+5}{6}}}{0.035(v+5)}$		$\alpha_d d_\infty$
	f	$\frac{1}{1+e^{(v+35)/9}}+\frac{0.6}{1+e^{(50-v)/20}}$	$\frac{1}{0.02e^{-(0.034(v+14.5)^2)}+0.02}$		α_f
	f_{Ca}	$\frac{1.7c_d}{1.7c_d+0.012}$	$\frac{1}{1.7c_d^{1.5}+0.012}$		α_{Ca}
I_{to}	q_{to}	$\frac{1}{1+e^{(v+53)/13}}$	$\frac{39}{0.57e^{-0.08(v+44)}+0.065e^{0.1(v+46)}}$	6	$\frac{\alpha_{q_{\text{to}}}+\beta_{q_{\text{to}}}}{(Q_{10}^{\text{to}})^{Q_p}}$
	r_{to}	$\frac{1}{1+e^{-(v-22.3)/18.75}}$	$\frac{14.4}{e^{0.09(v+30.61)}+0.37e^{-0.12(v+24)}}$	2.75	$\frac{\alpha_{r_{\text{to}}}+\beta_{r_{\text{to}}}}{(Q_{10}^{\text{to}})^{Q_p}}$
I_{Ks}	x_{Ks}	$\frac{1}{1+e^{-(v+3.8)/14}}$	$\frac{990}{1+e^{-(v+2.4)/14}}$		$\frac{\alpha_{x_{\text{Ks}}}}{(Q_{10}^{\text{Ks}})^{Q_p}}$
I_{Kur}	x_{Kur1}	$\frac{1}{1+e^{-(v+30.3)/9.6}}$	$\frac{0.65}{e^{-(v+10)/8.5}+e^{-(v-30)/59}}$	$\frac{0.65}{2.5+e^{(v+82)/17}}$	$\frac{1}{(\alpha_{x_{\text{Kr1}}}+\beta_{x_{\text{Kr1}}})(Q_{10}^{\text{Kur}})^{Q_p}}$
	x_{Kur2}	$\frac{1}{1+e^{(v-99.45)/27.48}}$	$\frac{1}{21+e^{-(v-185)/28}}$	$e^{(v-158)/16}$	$\frac{1}{(\alpha_{x_{\text{Kr2}}}+\beta_{x_{\text{Kr2}}})(Q_{10}^{\text{Kur}})^{Q_p}}$
I_{f}	x_{f}	$\frac{1}{1+e^{(v+78)/5}}$	$\frac{1900}{1+e^{(v+15)/10}}$		$\frac{\alpha_{x_{\text{f}}}}{(Q_{10}^{\text{f}})^{Q_p}}$

Table S9: Specification of the parameters z_∞ and τ_z, for $z = m$, j, m_L, h_L, d, f, f_{Ca}, q_{to}, r_{to}, x_{Ks}, x_{Kur1}, x_{Kur2}, and x_{f} in the equations for the gating variables (2).

S2 Supplementary figures

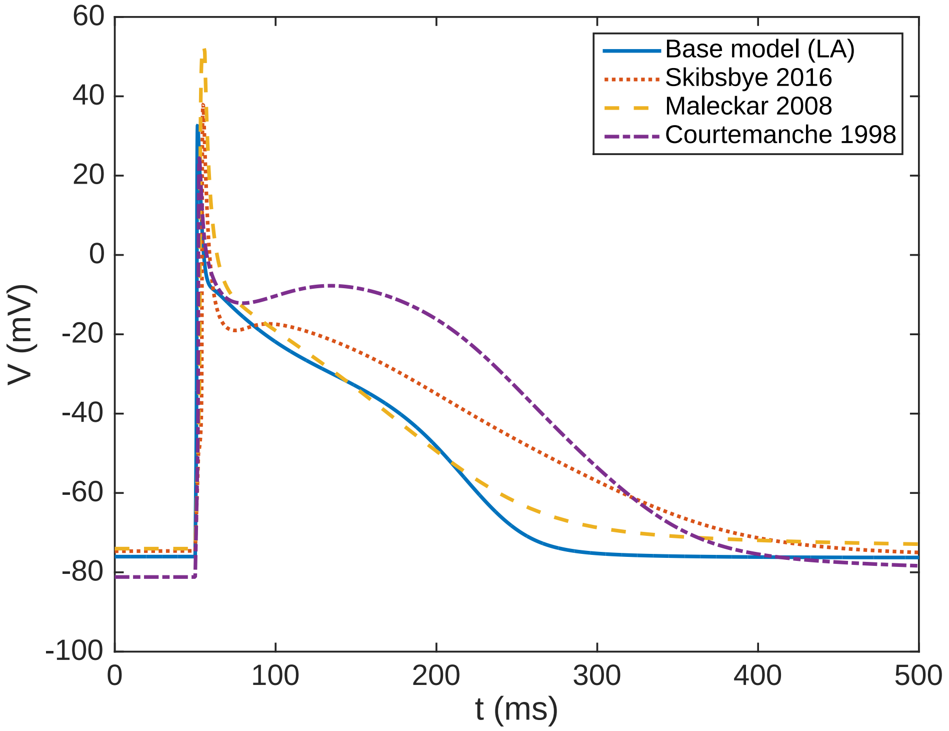

Figure S1: Comparison of the action potential of the LA version of the base model to other published models of the human atrial action potential, i.e., the Skibsbye et al. 2016 model [13], the Maleckar et al. 2008 model [12], and the Courtemanche et al. 1998 model [10]. All models are paced at 1 Hz.

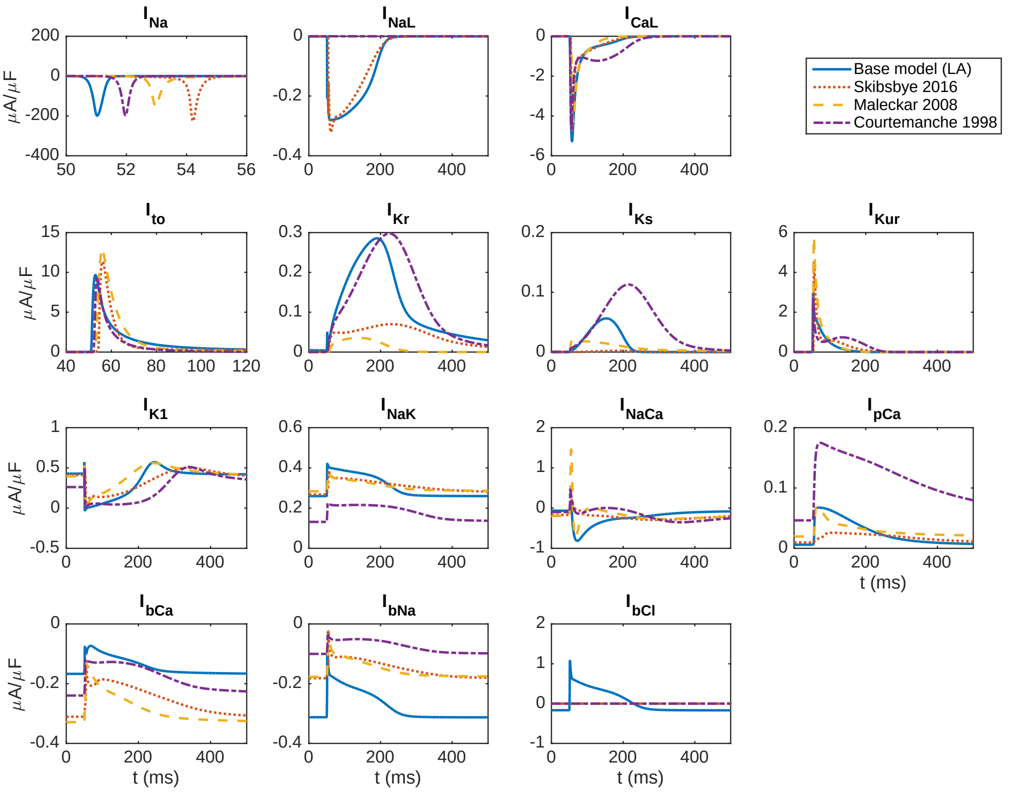

Figure S2: Comparison of the current densities during an action potential of the LA version of the base model to other published models of the human atrial action potential, i.e., the Skibsbye et al. 2016 model [13], the Maleckar et al. 2008 model [12], and the Courtemanche et al. 1998 model [10]. All models are paced at 1 Hz.

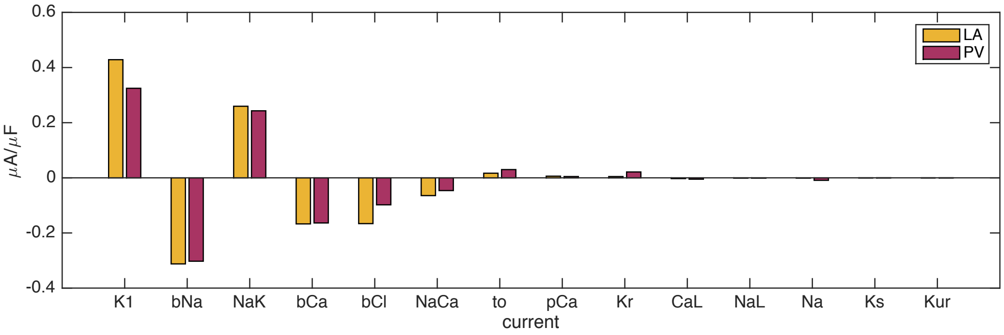

Figure S3: Current densities at rest for the LA and PV versions of the base model. More specifically, the currents are recorded 50 ms before the time of stimulation in a simulation with a 1 Hz pacing frequency.

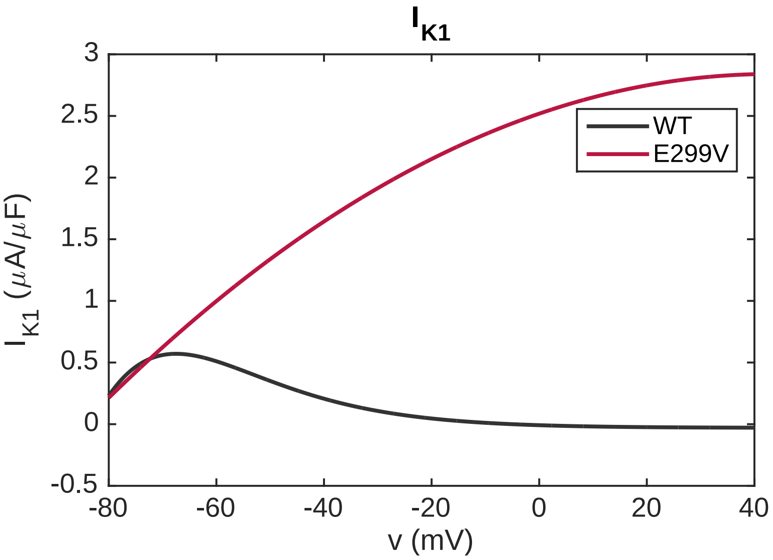

Figure S4: WT and E299V I_{K1} current densities as functions of the membrane potential, v.

S3 Wavelength and re-entry

According to the classical hypothesis, re-entry can only be sustained if the geometry of the substrate can accommodate the wavelength. An example of this is reported in Table 6 of the main text. However, we observe in this table that the wavelength is 4.9 cm for both the N588K and the E375X mutations, whereas the circumference of the cylinder of myocytes measures 4.7 cm. Although, according to the general rule, none of these mutations should be able maintain re-entry; we do observe re-entry for N588K and not for E375X (see Table 6 and Figure 8 and Figure 9 in the main text). This illustrates that (particularly when APD90 is used as proxy for ERP) the 'rule' mentioned above is best regarded as a useful guideline as opposed to a mechanistic principle. A more detailed analysis of the dynamic balance of currents (particularly the input resistance) during terminal repolarization is required to accurately distinguish conditions for re-entry. In order explain those dynamics, we have plotted the action potentials of WT, N588K and E375X (Figure S5, and associate currents in Figure S6) and monitored how they are perturbed if a small excitatory stimulus current (6 μA/μF for 3 ms) is added at various time points in repolarization. We observe that for WT, and both E375X and N588K, input resistance is sufficiently low that the perturbation readily decays if the stimulus current is applied earlier than 140 ms. However, when the stimulus current is applied after 150 ms, we see that the E375X and WT models have maintained low input resistance (small deviation in membrane potential), but for N588K input resistance has increased considerably to allow much larger stimulated depolarization. This in turn reflects that the N588K mutation would be more apt to undergo re-entrant excitation at this point in repolarization, and earlier than either E375X or WT could be re-excited. Often, effects of this type can be traced to enhanced recovery from inactivation of the sodium current, however, as shown in the lower left panel of Figure S5, the degree of inactivation up to $\sim$ 200 ms is relatively consistent for all of the models. Thus, we suggest it is unlikely that recovering I_{Na} is the differentiating mechanism. Instead Figure S6 shows a clear difference in I_{Kr} and I_{Ks} (and to a lesser extent I_{K1} and I_{NaK}) at this point in repolarization. This difference would permit much lower input resistance in both WT and E375X than N588K during this terminal phase of repolarization. Thus, we suggest that these differences in dynamic activation of the delayed rectifier K^+ currents are most likely to explain the greater susceptibility to re-entry in our N588K simulations.

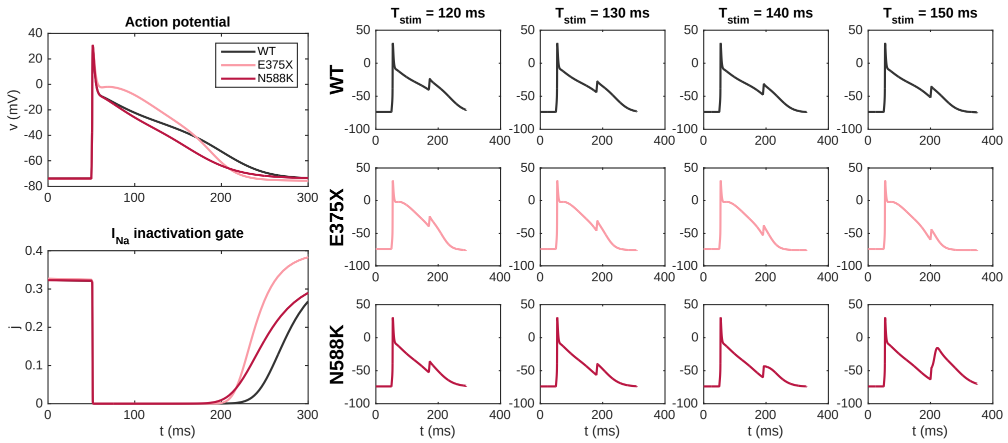

Figure S5: Upper left panel: The action potential for WT, and for the N588K and E375X mutations. We observe (see also Table 6 in the main manuscript) that APD90 is the same for the N588K and E375X mutations, but that earlier repolarization is considerably faster for N588K. Lower left panel: The value of the inactivation state variable for the I_{Na} currents during the action potentials in the upper panel. Right panels: We apply a stimulus current of 6 μA/μF for 3 ms at different points in time after the first stimulation, but before full reploarization is obtained. The values (T_{stim}) indicated in the column titles represent the time interval between the first and the second stimulation. For $T_{\mathrm{stim}} = 150$ ms, the perturbation caused by the stimulus current decays rapidly for WT and for the E375X mutation, but for the N588K mutation, a more pronounced depolarization is observed. Note that in these simulations, we consider a model representing the 'average' of the PV and LA models (all parameters are set to the mean of the PV and LA values).

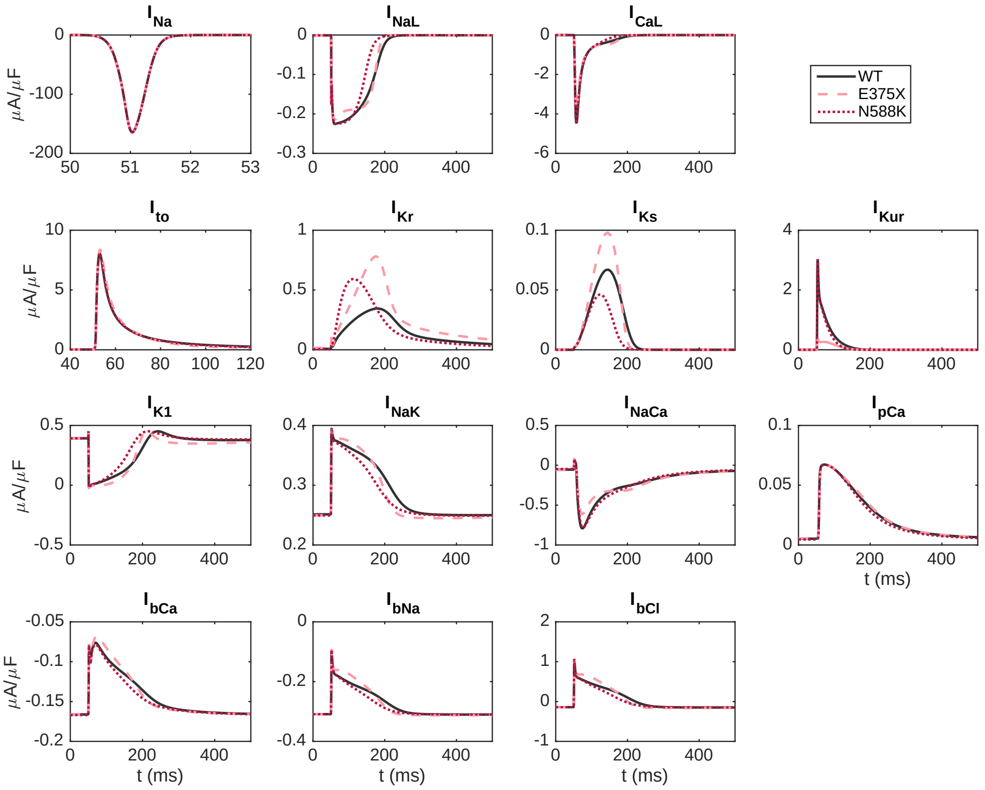

Figure S6: The membrane current densities for WT, and for the N588K and E375X mutations. We consider a model representing the 'average' of the PV and LA models (all parameters are set to the mean of the PV and LA values). The corresponding action potentials are plotted in the left panel of Figure S5.

References

[1] Karoline Horgmo Jæger, Verena Charwat, Bérénice Charrez, Henrik Finsberg, Mary M Maleckar, Sam Wall, Kevin Healy, and Aslak Tveito. Improved computational identification of drug response using optical measurements of human stem cell derived cardiomyocytes in microphysiological systems. *Frontiers in Pharmacology*, 10:1648, 2020.

[2] Karoline Horgmo Jæger, Samuel Wall, and Aslak Tveito. Computational prediction of drug response in short QT syndrome type 1 based on measurements of compound effect in stem cell-derived cardiomyocytes. *PLoS Computational Biology*, 17(2):e1008089, 2021.

[3] Eleonora Grandi, Francesco S Pasqualini, and Donald M Bers. A novel computational model of the human ventricular action potential and Ca transient. *Journal of Molecular and Cellular Cardiology*, 48(1):112–121, 2010.

[4] Thomas O'Hara, László Virág, András Varró, and Yoram Rudy. Simulation of the undiseased human cardiac ventricular action potential: Model formulation and experimental validation. *PLoS Computational Biology*, 7(5):e1002061, 2011.

[5] Michelangelo Paci, Jari Hyttinen, Katriina Aalto-Setälä, and Stefano Severi. Computational models of ventricular-and atrial-like human induced pluripotent stem cell derived cardiomyocytes. *Annals of Biomedical Engineering*, 41(11):2334–2348, 2013.

[6] Martin Fink, Denis Noble, Laszlo Virag, Andras Varro, and Wayne R Giles. Contributions of HERG K^+ current to repolarization of the human ventricular action potential. *Progress in Biophysics and Molecular Biology*, 96(1-3):357–376, 2008.

[7] Mark J McPate, Rona S Duncan, James T Milnes, Harry J Witchel, and Jules C Hancox. The N588K-HERG K^+ channel mutation in the 'short QT syndrome': mechanism of gain-in-function determined at 37 °C. *Biochemical and Biophysical Research Communications*, 334(2):441–449, 2005.

[8] Makarand Deo, Yanfei Ruan, Sandeep V Pandit, Kushal Shah, Omer Berenfeld, Andrew Blaufox, Marina Cerrone, Sami F Noujaim, Marco Denegri, José Jalife, and Silvia G Priori. KCNJ2 mutation in short QT syndrome 3 results in atrial fibrillation and ventricular proarrhythmia.

Proceedings of the National Academy of Sciences, 110(11):4291–4296, 2013.

[9] Eleonora Grandi, Sandeep V Pandit, Niels Voigt, Antony J Workman, Dobromir Dobrev, José Jalife, and Donald M Bers. Human atrial action potential and Ca^{2+} model: sinus rhythm and chronic atrial fibrillation. *Circulation Research*, 109(9):1055–1066, 2011.

[10] Marc Courtemanche, Rafael J Ramirez, and Stanley Nattel. Ionic mechanisms underlying human atrial action potential properties: insights from a mathematical model. *American Journal of Physiology-Heart and Circulatory Physiology*, 275(1):H301–H321, 1998.

[11] Ingrid E Christophersen, Morten S Olesen, Bo Liang, Martin N Andersen, Anders P Larsen, Jonas B Nielsen, Stig Haunsø, Søren-Peter Olesen, Arnljot Tveit, Jesper H Svendsen, and Nicole Schmitt. Genetic variation in KCNA5: impact on the atrial-specific potassium current I_{Kur} in patients with lone atrial fibrillation. *European Heart Journal*, 34(20):1517–1525, 2013.

[12] Mary M Maleckar, Joseph L Greenstein, Natalia A Trayanova, and Wayne R Giles. Mathematical simulations of ligand-gated and cell-type specific effects on the action potential of human atrium. *Progress in Biophysics and Molecular Biology*, 98(2-3):161–170, 2008.

[13] Lasse Skibsbye, Thomas Jespersen, Torsten Christ, Mary M Maleckar, Jonas van den Brink, Pasi Tavi, and Jussi T Koivumäki. Refractoriness in human atria: time and voltage dependence of sodium channel availability. *Journal of Molecular and Cellular Cardiology*, 101:26–34, 2016.

[14] Joachim R Ehrlich, Tae-Joon Cha, Liming Zhang, Denis Chartier, Peter Melnyk, Stefan H Hohnloser, and Stanley Nattel. Cellular electrophysiology of canine pulmonary vein cardiomyocytes: action potential and ionic current properties. *The Journal of Physiology*, 551(3):801–813, 2003.
